# Galectin-3 Mediated Inflammatory Response Contributes to Neurological Recovery by QiShenYiQi in Subacute Stroke Model

**DOI:** 10.3389/fphar.2021.588587

**Published:** 2021-04-19

**Authors:** Yule Wang, Shuang He, Xinyan Liu, Zhixiong Li, Lin Zhu, Guangxu Xiao, Xiaoli Du, Hongxia Du, Wen Zhang, Yiqian Zhang, John Orgah, Yuxin Feng, Boli Zhang, Yan Zhu

**Affiliations:** ^1^ State Key Laboratory of Component-based Chinese Medicine, Tianjin University of Traditional Chinese Medicine, Tianjin, China; ^2^ Research and Development Center of TCM, Tianjin International Joint Academy of Biotechnology and Medicine, Tianjin, China; ^3^ Pharmaceutical Informatics Institute, College of Pharmaceutical Sciences, Zhejiang University, Hangzhou, China; ^4^ Inner Mongolia Medical University, Jinshan Economic and Technological Development District, Inner Mongolia, China; ^5^ State Key Laboratory of Core Technology in Innovative Chinese Medicine, Beijing, China; ^6^ State Key Laboratory of Core Technology in Innovative Chinese Medicine, Tianjin Tasly Holding Group Co., Ltd., Tianjin, China

**Keywords:** qishenyiqi, cerebral ischemia/reperfusion injury, TMT-based quantitative proteomics analysis, inflammatory response, galectin-3

## Abstract

Effective therapies for stroke are still limited due to its complex pathological manifestations. QiShenYiQi (QSYQ), a component-based Chinese medicine capable of reducing organ injury caused by ischemia/reperfusion, may offer an alternative option for stroke treatment and post-stroke recovery. Recently, we reported a beneficial effect of QSYQ for acute stroke *via* modulation of the neuroinflammatory response. However, if QSYQ plays a role in subacute stroke remains unknown. The pharmacological action of QSYQ was investigated in experimental stroke rats which underwent 90 min ischemia and 8 days reperfusion in this study. Neurological and locomotive deficits, cerebral infarction, brain edema, and BBB integrity were assessed. TMT-based quantitative proteomics were performed to identify differentially expressed proteins following QSYQ treatment. Immunohistochemistry, western blot analysis, RT-qPCR, and ELISA were used to validate the proteomics data and to reveal the action mechanisms. Therapeutically, treatment with QSYQ (600 mg/kg) for 7 days significantly improved neurological recovery, attenuated infarct volume and brain edema, and alleviated BBB breakdown in the stroke rats. Bioinformatics analysis indicated that protein galectin-3 and its mediated inflammatory response was closely related to the beneficial effect of QSYQ. Specially, QSYQ (600 mg/kg) markedly downregulated the mRNA and protein expression levels of galectin-3, TNF-α, and IL-6 in CI/RI brain as well as serum levels of TNF-α and IL-6. Overall, our findings showed that the effective action of QSYQ against the subacute phase of CI/RI occurs partly *via* regulating galectin-3 mediated inflammatory reaction.

## Introduction

Stroke is recognized as one of the main leading causes of death and serious long-term disability, as well as cognitive functional impairment, worldwide (Benjamin et al., 2019). Of all strokes, ischemic stroke incidents account for approximately 87% (Benjamin et al., 2019). Although intensive basic and clinical research has uncovered multiple modifiable risk factors (such as high systolic blood pressure, high fasting plasma glucose, and high total cholesterol) (Feigin et al., 2016), and has revealed many potential molecular mechanisms (such as inflammatory response (Jin et al., 2013), oxidative stress (Li et al., 2018), blood-brain barrier dysfunction (Jiang et al., 2018), activation of apoptotic and autophagic pathways (Kalogeris et al., 2012), mitochondrial dysfunction (Yang et al., 2018), and complement activation of stroke (Ma et al., 2019), effective therapies are still limited. Currently, the standard clinical therapy for appropriate patients with acute ischemic stroke are tissue plasminogen activator (tPA)-mediated intravenous thrombolysis and executing intra-arterial thrombectomy to realize recanalization (Dong et al., 2017; Goda et al., 2020). However, the major problems of the relatively narrow therapeutic time window and a high risk of hemorrhagic transformation in tPA treatment should not be ignored (Dong et al., 2017; Puig et al., 2017). Reperfusion injury following the restoration of blood supply may also result in more adverse stroke outcomes *via* complicated pathological processes (Lin et al., 2016). It is believed that cerebral ischemia/reperfusion injury (CI/RI) has become an increasingly critical challenge for post-stroke recovery (Pan et al., 2007). Thus, it is crucial to discover novel and alternative therapies for ischemic stroke.

In China, herbal remedies have been historically applied in the treatment of stroke and stroke-associated symptoms based on the theory of traditional Chinese medicine (TCM) (Sun et al., 2015; Han et al., 2017a). Qishen Yiqi (QSYQ) formula, which is composed of *Astragalus membranaceus* (Fisch.) Bge. (Huangqi), *Salvia miltiorrhiza* Bge. (Danshen), *Panax notoginseng* (Burk.) F. H. Chen (Sanqi), and *Dalbergia odorifera* T. Chen (Jiangxiang), is a typical component-based Chinese medicine that was approved by the State Food and Drug Administration of China in 2003 for treatment of angina pectoris of coronary heart disease origin with Qi deficiency and blood stasis syndrome (Cao et al., 2017), and secondary prevention of myocardial infarction (Shang et al., 2013). Recently, the major active ingredients in QSYQ, such as astragaloside IV, calycosin, calycosin-7-O-β-D-glycoside, formononetin, salvianolic acid A, salvianolic acid B, tanshinone IIA, notoginsenoside R1, ginsenoside Rg1, and ginsenoside Rb1(Yu et al., 2017; Zhang et al., 2018), have been reported to have therapeutic potential in acute and subacute phases of ischemic stroke *via* various functional mechanisms, especially immune-inflammatory response related mechanism (Han et al., 2017b; Zhang et al., 2020). Furthermore, we have found the protective action of QSYQ against acute ischemic stroke *via* regulating neuroinflammatory network mobilization in a mouse model of cerebral ischemia and reperfusion (Wang et al., 2020).

At present, it is believed that immune-inflammatory response is an important endogenous mechanism involved in the pathophysiological process of CI/RI (Famakin, 2014). An anti-inflammatory strategy appears to be a favorite therapeutic target, given its pleiotropic roles in the acute damage to long term recovery phase of ischemic stroke (Peng et al., 2019). Galectin-3 is a unique chimera-type member of the galectin family and exerts various functions depending on cell type and cellular location (Yip et al., 2017). Recent studies suggested that galectin-3 may serve as a promising prognostic biomarker as well as a potential therapeutic target for cardiovascular and cerebrovascular diseases (Shen et al., 2016; Dong et al., 2018). Accumulated evidence has indicated that galectin-3 appeared to function as a significant regulator participating in the neuroinflammatory reaction caused by cerebral ischemia and reperfusion (Shin, 2013; Rahimian et al., 2018). Interestingly, Burguillos et al. reported that microglia-secreted galectin-3 acted as an endogenous ligand for Toll-like receptor 4 (TLR-4) activation and deteriorated typical TLR4-dependent inflammatory response after cerebral ischemia (Burguillos et al., 2015).

In the present study, we aimed to explore the pharmacodynamic effect of QSYQ against the cerebral injury induced by ischemic stroke in the subacute phase. Subsequently, tandem mass tag (TMT)-based quantitative proteomics analysis followed by experimental verification was executed to explain the galectin-3 mediated neuroinflammatory mechanism of QSYQ treatment in response to CI/RI of the subacute phase.

## Materials and Methods

### Drugs and Reagents

QSYQ (drug approval number: Z20030139; batch number: 20160604), which was supplied by Tasly Pharmaceutical Group Co., Ltd. (Tianjin, China), was prepared according to the ratio of Huangqi: Danshen: Sanqi: Jiangxiang oil = 148.01:70.35:70.35:11.97 and dissolved in ultrapure water to make a solution at concentrations of 15 mg/ml, 30 mg/ml, and 60 mg/ml for experiments. Nimodipine (Nim) (drug approval number: H14022821) was purchased from Yabao Pharmaceutical Group Co., Ltd. (Taiyuan, China). Chromatographic-grade methanol and acetonitrile were purchased from Merck (Darmstadt, Germany). Water was purified using the Millipore-Q water purification system (Millipore, Milford, MA, United States). Standards of tanshinol, calycosin-7-O-β-D-glycoside, protocatechualdehyde, formononetin, and rosmarinic acid were purchased from the National Institutes for Food and Drug Control of China (Beijing, China). Standards of lithospermic acid, ononin, and calycosin were purchased from Tianjin Shilan Technology Co., Ltd. (Tianjin, China). Standards of salvianolic acid A, salvianolic acid B, salvianolic acid D, salvianolic acid T, and salvianolic acid U were obtained from Tianjin Tasly Pharmaceutical Group Co., Ltd. (Tianjin, China). The purities of the above standards were more than 98%. 2, 3, 5-Triphenyl-2H-Tetrazolium Chloride (TTC) solution (2%, G3005), paraformaldehyde (4%, P1110), RIPA lysis buffer (R0020), PMSF (100mM, P0100), BCA Protein Assay Kit (PC0020–50T), and SDS-PAGE Gel Kit (P1200–50T) were purchased from Solarbio (Beijing, China). Evans Blue was purchased from Meilunbio (MB4680-5°g, Dalian, China). Isoflurane (Lot No. B506) was purchased from Ruiwode Lifescience Co., Ltd. (Shenzhen, China). Omnipaque (300 mg I/ml, drug approval number: H20000,595) was purchased from GE Pharmaceutical Co., Ltd. (Shanghai, China). Tandem mass tag (TMT) (6-plex) isobaric label reagents were purchased from Thermo Fisher Scientific (Waltham, MA, United States). Sodium dodecyl sulfate (SDS), DL-dithiothreitol (DTT), Iodoaceamide (IAA), Triethylamine borane (TEAB), and urea were obtained from Bio-Rad (Hercules, CA, United,States). Trypsin was obtained from Promega (Madison, WI). Tris was obtained from Sigma (St. Louis, MO, United States). Anti-Galectin-3 antibody was purchased from the Abcam Company (ab53082, Cambridge, United Kingdom). Anti-β-actin antibody was purchased from Cell Signaling Technology (8457S, Beverly, MA, United States). Goat anti-rabbit IgG H&L was purchased from Zhongshan Jingqiao Biotechnology, Inc. (ZB-5301, Beijing, China). Cytokine IL-6 and TNF-α ELLISA kits were obtained from Shanghai Zhuocai Biotechnology Co., Ltd. (Shanghai, China).

### High Performance Liquid Chromatography Analysis

Mixed standards were prepared as follows: all standards were weighed accurately and dissolved in 10 ml of methanol. Mixed standards were then filtered using a 0.45 μm filtration membrane before analysis. Samples were extracted as follows: One hundred fifty milligrams of QSYQ was accurately weighed and ultrasonically extracted with 10 ml of 50% (v/v) methanol by ultrasonication for 30 min. After replenishing the lost weight during extraction, samples were filtered using a 0.45 μm filtration membrane for HPLC analysis.

The HPLC analysis of QSYQ was performed using an Agilent 1,290 Infinity LC, which was equipped with a photodiode array ultraviolet-visible detector. A Waters ACQUITY UPLC BEH Shield RP18 column (2.1 × 100 mm, 1.7 µm) was used to perform the chromatography separation using water (A) and acetonitrile (B) as mobile phases. The gradient elution was set as follows: 0–3 min, 15% B; 3–10 min, 15–20% B; 10–12 min, 20% B; 12–18 min, 20–40% B; 18–24 min, 40–70% B; 24–27 min, 70–95% B; and 27–28 min, 95–8% B. The column temperature was maintained at 25°C with an injection volume of 2 μL and a flow rate of 0.4 ml/min for analysis. Dual-wavelength detection was applied in this analysis: channel A was set as 254 nm; channel B was set as 280 nm from 0 to 3 min and 325 nm from to 28 min.

### Experimental Animals

Male Sprague-Dawley (SD) rats weighing 200–220 g were obtained from Beijing Vital River Lab Animal Technology Co., Ltd. (Beijing, China, Certificate No: SCXK [Jing] 2014-0013). Rats were housed under a 12 h light/dark cycle in polypropylene cages, which were well ventilated, and there was a controlled in-house temperature of 22 ± 2°C as well as humidity of 40 ± 5%. Commercial rodent chow (Beijing Vital River Lab Animal Technology Co., Ltd.) and clean water were provided ad libitum. Before the surgery, animals were fasted for 12 h, but with free access to water. The behavioral assessment was executed during the rats’ active periods.

### Middle Cerebral Artery Occlusion Model and Drug Treatment

Before the surgery, rats were anesthetized with 4% isoflurane in 70% nitrous oxide (N_2_O)/30% oxygen (O_2_). Then, isoflurane was lowered to 2.5% to maintain anesthesia using a small animal anesthesia machine (Matrix VIP 3,000; Midmark, United States). Throughout all surgical procedures, the animal was placed on a heating pad to keep body temperature at 37°C. Surgery was performed using transient MCAO technique as described elsewhere with proper modification (Uluc et al., 2011; Gubskiy et al., 2018; Lopez and Vemuganti, 2018). A ventral midline incision (∼1 cm) was made at the sterile neck and the left common carotid artery (CCA), external cerebral artery (ECA), and internal cerebral artery (ICA) were orderly exposed. After temporarily blocking the left CCA and ICA using two microsurgical clips, two sterile 4–0 silk sutures were placed around the left ECA: one tight ligature was tied as distally as possible from the bifurcation and the other loose ligature was put near the bifurcation. Then, a small hole was created between two silk sutures on the left ECA with a microsurgical scissor and a 3.0–5.0 cm length of silicone-coated 4-0 nylon monofilament (diameter with coating 0.32 ± 0.02 mm, Guangzhou Jialing Biotechnology Co., Ltd., Guangzhou, China) was inserted into the left ECA and gently guided toward the ICA. The microsurgical clip from the ICA was removed and advanced toward the monofilament until the tip occluded the origin of the left middle cerebral artery (MCA), resulting in a decline of local cerebral blood flow to 20% of baseline. After 90 min MCAO, the perfusion of blood flow was regained by withdrawing the monofilament. Before its closing, the wound area was moisturized with sterile saline and lidocaine was applied as a topical analgesic. Isotonic saline was intraperitoneally injected to prevent dehydration. After wound closure, bupivacaine was applied along the sutures and the animal was placed in a temperature-controlled recovery chamber to monitor its behavior for 1–2 h. Finally, the rat was put back to the housing cage and moistened rodent chow was placed on the bottom of the cage to facilitate eating. Sham-operated rats were handled in the same way like the model group, although the monofilament was not used to achieve the occlusion of MCA.

### Animal Grouping and Drug Treatment

After 22.5 h reperfusion, two independent observers, who were blinded to the experiment, tested the neurological deficits of the animals that had undergone MCAO surgery according to a five-point scale described previously by Longa et al. with a minor modification (Longa et al., 1989). The criteria were set as follows: score 0, no neurological deficit; score 1, mild focal neurological deficit (failure to fully extend contralateral forelimb); score 2, moderate focal neurological deficit (repetitive circling to the contralateral side); score 3, severe focal neurological deficit (falling to the contralateral side); and score 4, unable to walk spontaneously with a depressed level of consciousness or death. A total of 130 rats with a score between 1 and 2 were selected for the present study. As shown in Supplementary Figure S1, apart from the Sham group, the selected experimental rats were randomly divided into five different groups, including I/R (model), Nim (positive control)+I/R, QSYQ low dose (150 mg/kg) + I/R, middle dose (300 mg/kg) + I/R, and high dose (600 mg/kg) + I/R groups. Rats in QSYQ (150 mg/kg, 300 mg/kg, 600 mg/kg) and Nim (12 mg/kg) groups were orally administered their respective dose once daily for 7 days. Meanwhile, the Sham-operated and I/R model groups were given 0.9% normal saline via gavage at a dose of 10 ml/kg.

### Neurological Deficits and Mortality Rate

For the evaluation of neurological function, a modified Neurological Severity Score (mNSS) was used on days 1, 3, 5, and 7, respectively after MCAO. The mNSS was a synthetic scale, including motor function, sensory disturbance, balance, and reflection tests, graded on a score of 0–18 (Chen et al., 2001). The details were shown in Supplementary Table S1. One point was defined as inability to complete the tasks or no response to tests, while higher scores represented more serious neurological deficit. All tests were assessed by two independent investigators who were blinded to the experimental groups. The mortality rate of each group was calculated for the whole period of the drug treatment.

### Behavioral Assay

Open field test was executed to evaluate the locomotor activity of experimental animals (Jin et al., 2015). On the 8th day after stroke, the rats were respectively placed in four open field locomotion chambers (50 cm in length× 50 cm in width× 40 cm wall height) made of plywood. The bottom of the chamber was actually divided by white drawn lines, composing nine equal-area grids. After 5 min adaption to the novel environment, the locomotor data of each rat was automatically recorded for 30 min and calculated using the ANY-maze software (version 4.82, Stoelting, United States). The following parameters were analyzed: total distance traveled by the animals and average speed. The behavioral assessment was performed in a quiet room, from 8:00–12:00 a.m., by two observers who were blinded to the animal experiment.

### Gait Analysis

For the gait test, CatWalk XT 9.1 (Noldus Information Technology, Wageningen, Netherlands), an automated quantitative gait analysis system, was performed in this study (Orgah et al., 2019). Prior to surgery, rats were trained for at least three consecutive days to adapt to the walkway in a quiet room. On the 8th day after the MCAO or sham-operation, post-surgery testing was executed in the same conditions as the training period. Each experimental animal was placed individually on the runway and allowed to freely run back and forth until three accepted runs were recorded. The subsequent data analysis was handled using the Catwalk XT 9.1 Software. The main gait parameters, including walking speed (the speed of rats across the runway), base of support (distance between girdle paw pairs), stride length (distance the paw traveled from one step to the next), and stand (time duration of the paw in contact with the floor during a step cycle), were employed for assessing the effects of QSYQ on functional recovery. At least two observers who were blinded to the animal grouping carried out the CatWalk test and data analysis.

### Micro-CT Imaging and Analysis

After 8 days post-reperfusion, anesthetized rats were intra-arterially injected with a clinically available iodinated x-ray contrast agent (Omnipaque) at 2.0 ml over a period of 20 s. Subsequently, the micro-Computed Tomography (CT) imaging technique was used to visualize the blood-brain barrier (BBB) disruption and cerebral edema (Park et al., 2014; Orgah et al., 2018). In brief, brain micro-CT imaging was performed using a small animal micro-CT imager (Quantum FX; PerkinElmer, United States) with the following main imaging parameters: voltage for 90 kV, current for 180 μA, field of view (FOV) for 40 mm, and scan for 4.5 min. BBB disruption was detected for Omnipaque leakage and cerebral edema was presented with position offset of midline. Next, the “VOL EDIT” module in Analyze 12.0 image analysis software (Analyze Direct, Overland Park, KS, United States) was applied to make 3D reconstructions of whole-cerebral slide images. The “ROI” measure module was used to calculate the mean micro-CT number (HU) of Omnipaque leakage and cerebral hemisphere volumes.

### TTC Staining and Quantification of Infarct Volume

After performing micro-CT imaging, brains were quickly removed. Six pieces of 2 mm-thick coronal cerebral slices were obtained using a rat brain matrix (Shenzhen RWD life technology Co., Ltd., Guangzhou, China) and stained with 2% TTC solution in the dark for 10 min at 37°C. After stained cerebral slices were photographed, ImageJ image processing software (ImageJ 1.51, Wayne Rasband, National Institutes of Health, United States) was applied to measure cerebral infarct area. Ratios of infarct volume were displayed as a percentage (%) of (total cerebral infarct volume/total brain volume) ×100 (Lin et al., 1993).

### Assessment of BBB Permeability

BBB permeability was examined by the extravasation of Evans Blue (EB) stain into the brain following the tail-vein injection. After 7 days post-reperfusion, rats were injected with 4% EB solution in 0.9% normal saline (2 ml/kg) via the tail vein. 24 h later, the brains suffering perfusion were cut into 2 mm-thick coronal slices. After recording the EB-stained cerebral sections, the Caliper IVIS Lumina K Series III system was used for fluorescent imaging detection [58]. Then, the Living Image® Software (version 4.3.1) was used to quantify the EB leakage via calculating a total radiant efficiency [(p/s)/(μW/cm2)].

### TMT-Based Quantitative Proteomics Analysis

#### Sample Preparation

According to the above experimental results, rats from the model group (n = 3) and QSYQ high dose group (n = 3) were screened to further perform proteomic analysis. A total of eight days after MCAO, anesthetized rats were transcardially perfused with prechilled saline and then the brains were immediately removed. The infarcted cerebral hemisphere tissues were separated, snap frozen in liquid nitrogen, and stored at -80°C until use. Protein extraction from the cerebral tissues were carried out referring to a method previously described (Zhu et al., 2014) with some modifications. In brief, all samples were homogenized in ice-cold lysis buffer. Subsequently, the homogenate was sonicated and boiled for 15 min. After centrifugation at 14,000 g for 40 min at 4°C, the supernatant was filtered with 0.22 µm filters. Protein content was determined using the BCA protein assay kit.

### Protein Digestion Based on Filter-Aided Sample Preparation

Protein digestion was performed according to the method of filter-aided sample preparation (FASP) (Wisniewski et al., 2009). 200 μg of proteins for each sample were incorporated into 30 μL SDT buffer (4% SDS, 100 mM DTT, 150 mM Tris-HCl pH 8.0). The detergent, DTT, and other low-molecular-weight components were removed using UA buffer (8 M Urea, 150 mM Tris-HCl pH 8.0) by repeated ultrafiltration (Microcon units, 10 kD). Then 100 μL iodoacetamide (100 mM IAA in UA buffer) was added to block reduced cysteine residues and the samples were incubated for 30 min in the dark. The filters were washed with 100 μL UA buffer three times and then 100 μL100 mM TEAB buffer twice. Finally, the protein suspensions were digested with 4 μg trypsin (Promega) in 40 μL TEAB buffer overnight at 37°C, and the resulting peptides were collected as a filtrate. The peptide content was estimated by UV light spectral density at 280 nm using an extinctions coefficient of 1.1 of 0.1% (g/L) solution that was calculated on the basis of the frequency of tryptophan and tyrosine in vertebrate proteins.

### TMT Labeling and Peptide Fractionation

According to the manufacturer’s instructions, 100 μg peptide mixture of each sample was labeled using tandem mass tags (TMT) isobaric label reagents (Thermo Fisher Scientific). Subsequently, pierce high pH reversed-phase peptide fractionation kit (Thermo Fisher Scientific, Waltham, MA, United States) was used to fractionate TMT-labeled digest samples into 15 fractions by an increasing acetonitrile step-gradient elution according to instructions (Wei et al., 2018). The fractions were dried, lyophilized, and stored at -80 C until LC-MS/MS analysis.

### Reversed-Phase Liquid Chromatography-Tandem Mass Spectrometry

The NanoLC-MS/MS analysis of each fraction was performed using a Q Exactive mass spectrometer (Thermo Fisher Scientific) that was coupled to an Easy nLC1000 HPLC system (Thermo Fisher Scientific). The labeled peptides were loaded onto a reverse phase trap column (Thermo Scientific Acclaim PepMap100, 100 μm inner diameter× 2 cm, nanoViper C18) connected to the C18-reversed phase analytical column (Thermo Scientific Easy Column, 10 cm long, 75 μm inner diameter, 3 μm resin) in buffer A (0.1% formic acid) and eluted with a 60 min linear gradient - 0–50% buffer B (84% acetonitrile and 0.1% formic acid), 50 min; 50–100% buffer B, 5 min; 100%–100% buffer B, 5 min - at a flow rate of 300 nL/min controlled by IntelliFlow technology. The mass spectrometer was operated in positive ion mode. MS data was acquired using a data-dependent top 10 method dynamically choosing the most abundant precursor ions from the survey scan (300–1800 m/z) for high-energy collision dissociation (HCD) fragmentation. Automatic gain control (AGC) target value was set to 3 × 10^6^ with a maximum ion injection time of 10 ms. Dynamic exclusion duration was 40.0 s. The survey scans were acquired at a high resolution of 70,000 at m/z 200, resolution for HCD spectra was set to 17,500 at m/z 200, and isolation width was 2 m/z. Normalized collision energy was 30 eV and the underfill ratio, which specified the minimum percentage of the target value likely to be reached at maximum fill time, was defined as 0.1%. The instrument was run with peptide recognition mode enabled.

### Protein Identification and Quantification

The MS/MS spectra were searched using MASCOT engine (Matrix Science, London, United Kingdom; version 2.2) embedded into Proteome Discoverer 1.4 (Thermo Fisher Scientific). Trypsin was chosen as the enzyme, allowing up to two missed cleavage sites. Carbamidomethylation (C), TMT 6-plex (N-term), and TMT 6-plex (lysine, K) were chosen as fixed modification. Oxidation (methionine, M) was regarded as a variable modification. The peptide mass tolerances were set at 20 ppm for MS1 spectra acquired, and the fragment mass tolerance for MS2 spectra was set to 0.1 Da. In this study, only rank 1 peptide and a false discovery rate (FDR) of ≤1% were accepted. The protein ratios were calculated as the median of only unique peptides of the protein. All peptide ratios were normalized by the median protein ratio, and the median protein ratio should be 1 after the normalization. Comparisons of the protein identification and quantitation results were done between each QSYQ high dose (H) and the corresponding model (M) groups. Significantly regulated proteins between experimental groups were determined based on their *p*-value (*p*-value<0.05). Only proteins with more than 1.20-fold or less than 0.833-fold change compared to control groups were considered differentially regulated.

### Bioinformatics Analysis

In this study, Cluster 3.0 (http://bonsai.hgc.jp/∼mdehoon/software/cluster/software.htm) and Java Treeview software (http://jtreeview.sourceforge.net) were used to perform hierarchical clustering analysis. Euclidean distance algorithm for similarity measure and average linkage clustering algorithm (clustering uses the centroids of the observations) for clustering were selected when carrying out hierarchical clustering. The acquired protein relative expression data was visualized as a tree heat map. Then, the data, including significantly differential protein name, *p*-value, and log(fold change), were imported into Ingenuity® Pathway Analysis (IPA) system (http://www.ingenuity.com) to execute further analysis. “Core analysis-Diseases and Functions” module was performed to obtain the top diseases and functions related to the significantly differential proteins. “Build-Connection” module was used to analyze protein-protein interaction (PPI) network.

According to the direct functional degree of each protein, the importance of the protein in the PPI network was displayed. “Path designer” module was used to polish the network images and graphs.

The algorithm of the IPA analysis was based on Fisher’s exact test with the enrichment score of *p*-value.

### Immunohistochemistry Analysis

After 8 days post-reperfusion, brain tissues were quickly obtained from euthanized rats and then fixed in 4% paraformaldehyde for at least 48 h. The fixed tissues were dehydrated and embedded in paraffin blocks to be cut into 5 μm cerebral coronal paraffin slices. Subsequently, the sections were dewaxed in xylene and rehydrated followed by incubation with 3% hydrogen peroxide at room temperature for 10 min to block intrinsic peroxidase activity. After performing antigen repair, the brain slices were treated with blocking buffer (10% bovine serum) at 37 C for 1 h to block any nonspecific antibody responses. Next step, the sections were incubated overnight at 4 C using anti-galectin-3 antibody diluted with blocking buffer (1:50), then sequentially incubated with secondary antibody (the biotin-conjugated goat anti-rabbit IgG) diluted with blocking buffer (1:200) for 40 min at 37 C. The immunoreactivity of galectin-3 protein was visualized using a DAB substrate kit, followed by counterstaining hematoxylin, differentiation with 1% hydrochloric acid alcohol, dehydration, and sealing. Finally, the optical microscope (Vectra 3, PerkinElmer, United States) was carried out to observe and photograph the brain slices. ImagePro Plus software (National Institutes of Health, Bethesda, MD, United States) was executed to quantify the expression of galectin-3 by calculating the average optical density (AOD) value.

### Western Blotting Analysis

The frozen post-ischemic hemisphere tissues (n = 3 per group) were collected in ice-cold RIPA lysis buffer containing serine proteases and acetylcholinesterase inhibitors. The lysates were centrifuged at 12,000 g for 10 min at 4 C. The BCA protein assay kit was used to measure the protein concentration. Equal amounts of 30 μg protein extracts were loaded and separated by 12% sodium dodecyl sulfate-polyacrylamide gels (SDS-PAGE) and transferred to polyvinylidene fluoride (PVDF) blotting membrane (GE Healthcare, Millipore, United States) using an electrophoresis apparatus (Tanon Science and Technology Co., Ltd., Shanghai, China). The membrane was blocked with a 5% non-fat milk-Tris-HCl-buffered saline and Tween 20 (TBST) solution for 3 h at room temperature, and then incubated overnight at C with the antibodies of anti-Galectin-3 (1:1,000) and anti-β-actin (1:2,000). After washing three times with TBST, the membrane was incubated with goat anti-rabbit IgG H&L (1:10,000) as the secondary antibody for 2 h at room temperature. After incubation finished, the membrane was washed three times (10 min each) and visualized using enhanced chemiluminescence detection reagents (TransGen Biotech Co., Ltd., Beijing, China). Western blotting bands were captured using the C-DiGit scanner with Image Studio (Version 5.2) imaging system. The quantification of band intensity was performed according to integrated density by ImageJ image processing software (ImageJ 1.42, Wayne Rasband, National Institutes of Health, United States).

### Quantitative Real-Time Polymerase Chain Reaction Analysis

Total RNA samples from the post-ischemic hemisphere tissues were isolated using TRIzol® reagent (Invitrogen, Thermo Fisher Scientific, Inc., Waltham, MA, United States) according to the manufacturer’s protocol. Subsequently, the complementary DNA (cDNA) was synthesized from 1 µg total RNA in a 20 µL reaction using Transcriptor First Strand cDNA Synthesis Kit (Roche, Mannheim, Germany).

The Bestar®Sybr Green qPCR Master Mix (DBI®Bioscience, Ludwigshafen, Germany) was used in reverse transcription-quantitative real-time polymerase chain reaction (RT-qPCR) to quantify the mRNA expression levels of LGALS3 (galectin-3), TNF-α, and IL-6 with glyceraldehyde 3-phosphate dehydrogenase (GAPDH) as an internal control. The designed gene-specific primers were obtained by Sangon Biotech (Shanghai, China) and the following oligonucleotide primer sequences were used: for LGALS3 forward 5′-GAG AAC AAC AGA AGA GTC ATC GTG-3′, reverse 5′-GAC CTG TAT TTT GAA TGG TTT GCC-3′; for TNF-α forward 5′-GCG TGT TCA TCC GTT CTC TA-3′, reverse 5′-CGT CTC GTG TGT TTC TGA GC-3′; for IL-6 forward 5′-ACC TGG AGT TTG TGA AGA ACA AC-3′, reverse 5′-GGA AGT TGG GGT AGG AAG GA-3′; for GAPDH forward 5′-GGC CTT CCG TGT TCC TAC C-3′, reverse 5′-CGC CTG CTT CAC CAC CTT C-3′.The amplification and analysis were performed using LightCycler®480 Software Version1.5.0.39 (Roche, Mannheim, Germany) for 45 cycles. The relative mRNA expression levels were calculated using 2−ΔΔCT method, following normalization to the housekeeping gene GAPDH.

### Enzyme-Linked Immunosorbent Assay

After 8 days post-reperfusion, the blood samples were collected from anesthetized rats. Then, blood samples were centrifuged at 10,000 rpm for 15 min at 4 C to separate and obtain the serum. In order to measure the expression levels of TNF-α and IL-6 in serum, the rat TNF-α ELISA kit (ZC-37624, ZCi Biotechnology Co., Ltd.) and rat IL-6 ELISA kit (ZC-36404, ZCi Biotechnology Co., Ltd.) were applied according to the manufacturer’s instructions. Finally, the protein concentrations of TNF-α and IL-6 in serum were calculated with the reference to the standard curve acquired from a gradient concentration standard substances provided by the assay kit.

Statistical analysis Statistical analysis was performed using Student’s two-tailed t-test for comparison between two groups and using one-way analysis of variance (ANOVA) followed by Dunnett’s t-test for comparisons between multiple groups. The mortality was analyzed using the Kaplan-Meier survival curve. A value of *p*< 0.05 was defined as a statistically significant difference. Data from different experiments were expressed either as mean ± SD or mean ± SEM as indicated. GraphPad Prism 7 software (GraphPad Software, Inc., La Jolla, CA, United States) was used to generate all data graphs.

## Results

### Chemical Profile of Major Ingredients in QSYQ by HPLC

To elucidate the main chemical components in QSYQ, HPLC analysis was performed in this study. A representative HPLC chromatogram was displayed in Supplementary Figure S2. Thirteen constituents in QSYQ were successfully identified according to the comparison of the retention times with standard substances, which was similar with the previous report (Zhang et al., 2018).

### QSYQ Improved the Survival Rate and Neurological Scores of Subacute Stroke Rats

**FIGURE 1:**
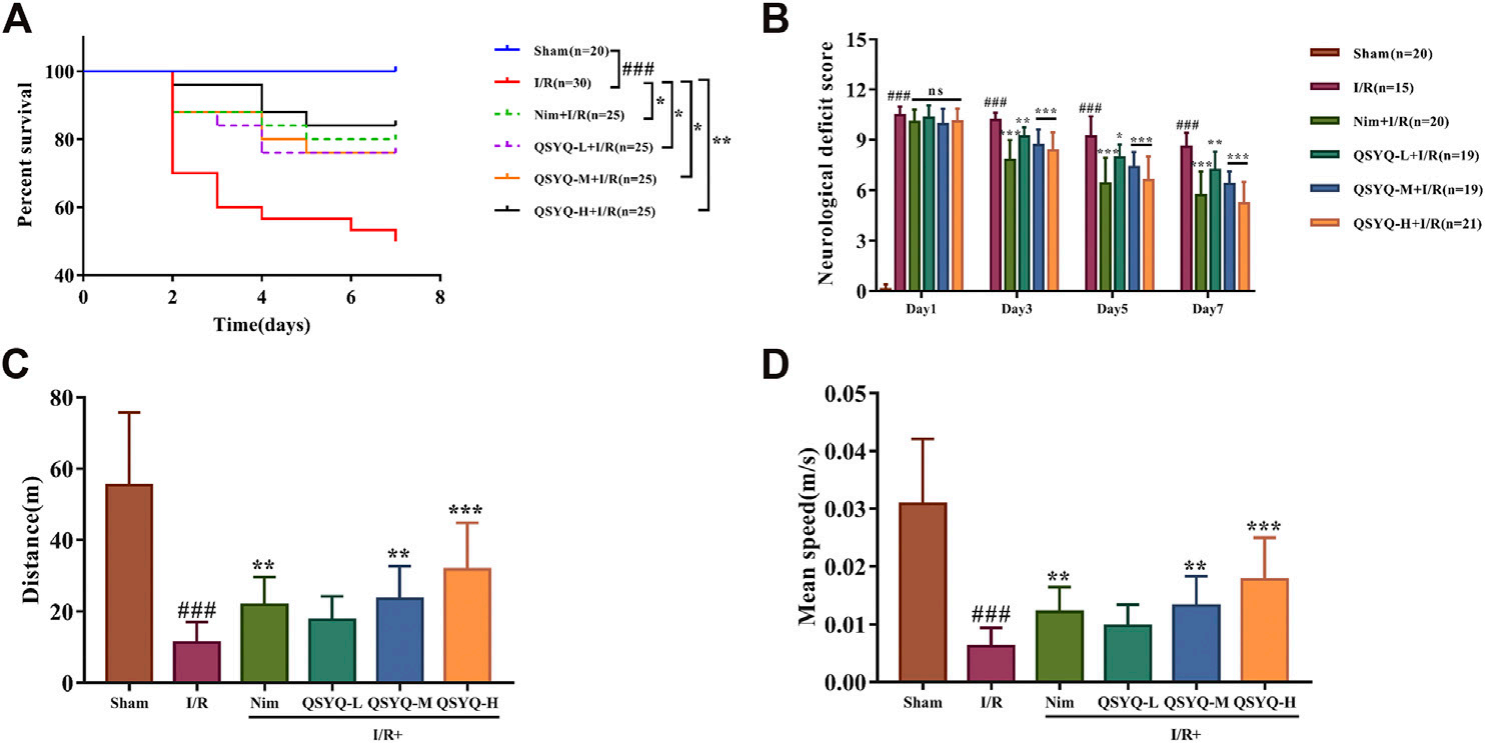
Effect of treatment with QSYQ on survival rate and neurological as well as motor deficits caused by stroke. **(A)** The Kaplan-Meier survival curve of each group. Treatment with QSYQ at all doses or Nim improved the survival rate compared to the model group. **(B)** The mNSS scores on days 1, 3, 5, and 7 after I/R surgery. The model group exhibited serious neurological deficit among six groups on days 3, 5, and 7 after drug treatment, whereas QSYQ and positive control groups showed a distinct reduction in mNSS scores. **(C)** Bar graph representing results of traveled total distance (n = 15). QSYQ at middle and high doses/Nim + I/R groups obviously improved the total traveled distance compared to the stroke rats. **(D)** Bar graph representing results of mean speed (n = 15). QSYQ at middle and high doses/Nim + I/R groups significantly boosted the mean speed compared to the stroke rats. Data are expressed as the mean ± SD. #*p*< 0.05, ##p < 0.01, and ###*p*< 0.001 vs. the sham group; *p< 0.05, **p< 0.01, and ***p< 0.001 vs. the model group. I/R, ischemia/reperfusion; Nim, Nimodipine; QSYQ-L, QSYQ 150°mg/kg-low dose; QSYQ-M, QSYQ 300°mg/kg-middle dose; QSYQ-H, QSYQ 600°mg/kg-high dose.

The Kaplan-Meier survival curve showed the survival proportion of the stroke-induced rats (Figure 1A). Rats after MCAO significantly increased mortality, especially during the acute period of reperfusion (first 72 h), compared to the sham group within the 7 day period of data collection. As expected, MCAO rats treated with low and middle doses of QSYQ or Nim obviously improved their survival rate (*p*<0.05). Furthermore, treatment with QSYQ at high doses (600 mg/kg) increased the survival rate from 50 to 84% (*p*<0.01; Figure 1A). As shown in Figure 1B, rats subjected to sham operation behaved normally without neurological deficit symptoms, however, the left side cerebral I/R surgery caused serious neurological deficit of rats in the model group. Compared with the model group, treatment with Nim or different doses of QSYQ markedly decreased scores with better neurological function. Moreover, QSYQ at middle and high doses had similar effects on neurological function to the Nim group (Figure 1B). In addition, we also carried out an open field test to measure the locomotor activity of experimental animals on post-stroke day 8. The data of total distance traveled by the animals and average speed within the 30 min indicated severe motor deficits in the model group (Figures 1C,D). Fortunately, the Nim, QSYQ middle dose (300 mg/kg), and QSYQ high dose (600 mg/kg) groups significantly enhanced the locomotor activity of stroke rats (*p*<0.01).

### QSYQ Ameliorated Motion Deficits of Subacute Stroke Rats

**FIGURE 2:**
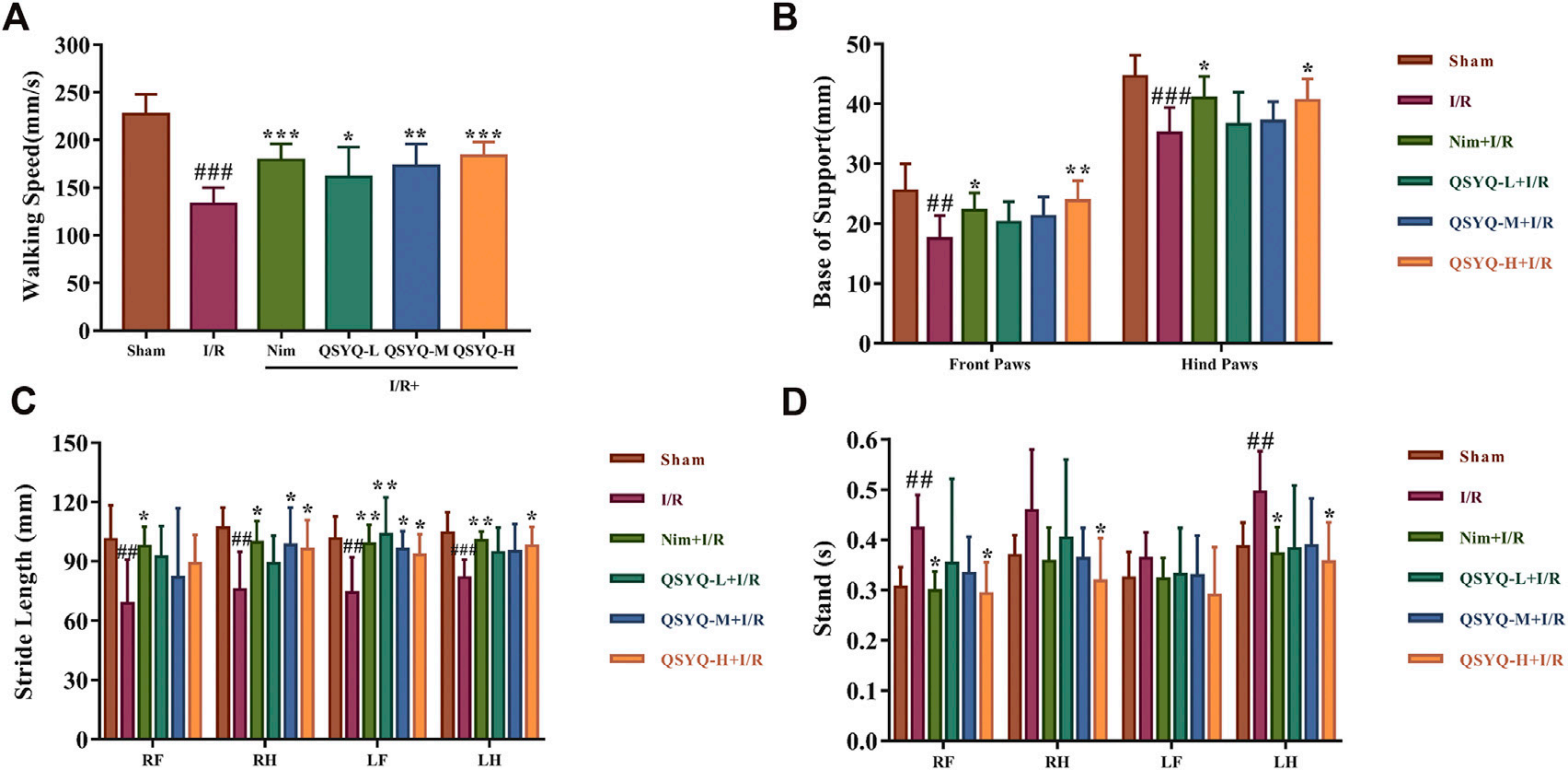
Locomotive gait analysis and functional recovery of QSYQ treated subacute stroke rats. **(A–D)** Bar graph representation of main parameter statistics, including walking speed, BOS, stride length, and stand time, for estimating functional recovery of QSYQ-treated stroke rats (n = 8). Left brain lesion induced by I/R resulted in a significant motion deficits of rats. Fortunately, QSYQ treatment could attenuate the abnormal motion function during the 8th-days post-reperfusion. Data are presented as the mean ± SD. #*p*< 0.05, ##*p*< 0.01, and ###*p*< 0.001 vs. the sham group; *p< 0.05, **p< 0.01, and ***p< 0.001 vs. the model group. I/R, ischemia/reperfusion; Nim, Nimodipine; QSYQ-L, QSYQ 150°mg/kg-low dose; QSYQ-M, QSYQ 300°mg/kg-middle dose; QSYQ-H, QSYQ 600°mg/kg-high dose.

In this study, we applied CatWalk XT 9.1 system to conduct the gait test of rats in each group 8 days after the MCAO or sham-operation. The collected footprints of rats from each group were automatically labeled by the system as right front paw (RF), right hind paw (RH), left front paw (LF), and left hind paw (LH). Once auto-classification of labeled footprints was finished, we could obtain the corresponding gait parameters of each labeled paw. Then, a one-way between subjects ANOVA was used to compare the effect of drugs on the function recovery of post-stroke motion impairment for walking speed, base of support (BOS), stride length, and stand time. In subacute phase of stroke, the rats subjected to MCAO significantly reduced walking speed, BOS, and stride length as well as increased stand time (Figure 2). Surprisingly, treatment with QSYQ could dose-dependently enhance walking speed (Figure 2A). Secondly, MCAO rats treated with QSYQ at high dose (600 mg/kg) obviously increased BOS of their front paws and hind paws (Figure 2B).

For the parameter of stride length, treatment with QSYQ at high dose (600 mg/kg) remarkably improved the impairment of RH, LF, and LH caused by stroke, while treatment with QSYQ at middle dose (300 mg/kg) only increased stride length in RH and LF (Figure 2C). Treatment with high doses of QSYQ positively reduced stand time of RF, RH, and LH (Figure 2D). As expected, treatment with Nim also obviously improved the deficit of locomotor function induced by cerebral ischemia and reperfusion (Figure 2).

### QSYQ Reduced Cerebral Infarction Volume and Cerebral Edema in Subacute Stroke Rat Brain

**FIGURE 3:**
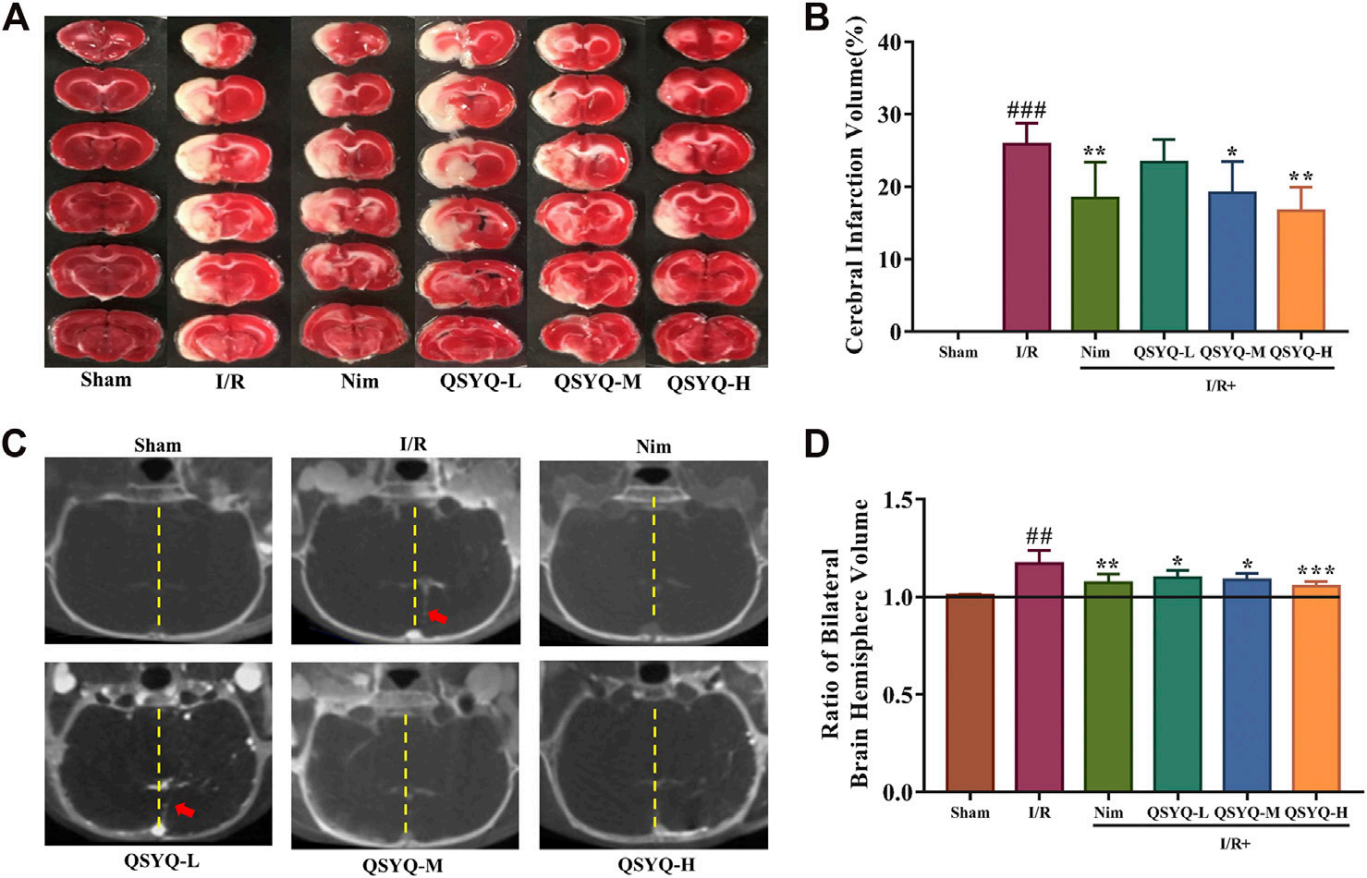
Effect of treatment with QSYQ on cerebral infarction and cerebral edema in subacute stroke rat brain. **(A)** Representative images of TTC-stained brain slices of each group. The infarct areas were in white, whereas normal areas were in red. **(B)** Quantitative analysis of cerebral infarction volumes of each group (n = 6). QSYQ/Nim + I/R group significantly decreased the infarct volumes caused by MCAO. **(C)** Representative coronal cerebral slice image of each group. Yellow dotted line marks the cerebral midline and red arrows indicate offset distance of midline. **(D**) Quantitative analysis of the ratio of bilateral brain hemisphere volume of each group (n = 6). Treatment with QSYQ at three doses as well as Nim decreased the raised values of the ratio of bilateral brain hemisphere volume due to left cerebral I/R injury. TTC staining and cerebral edema data are presented as the mean ± SD. ^**#**^ *p*< 0.05, ^**##**^ *p*< 0.01, and ^**###**^ *p*< 0.001 vs. the sham group; ^*^ *p*< 0.05, ^**^ *p*< 0.01, and ^***^ *p*<0.001 vs. the model group. I/R, ischemia/reperfusion; Nim, Nimodipine; QSYQ-L, QSYQ 150°mg/kg-low dose; QSYQ-M, QSYQ 300°mg/kg-middle dose; QSYQ-H, QSYQ 600°mg/kg-high dose.

By calculating the cerebral infarction volume in TTC-stained slices and indirectly assessing the severity of brain edema in micro-CT imaging, the brain injury in different groups on the 8th day after MCAO surgery was evaluated. Compared with the sham-operated group, the infarct volume was significantly increased in the experimental ischemic stroke model group (25.87 ± 2.88% of the cerebral volume), whereas treatment with Nim, QSYQ middle dose (300 mg/kg), and high dose (600 mg/kg) could exhibit a positive effect on reducing infarct volume induced by I/R injury (Figures 3A,B). According to the cerebral coronal image, we found that the model group presented the most obvious midline offset caused by cerebral edema, however, treatment with QSYQ at all three doses as well as Nim lessened the degree of midline offset (Figure 3C). Subsequently, Analyze 12.0 image analysis software was used to calculate the cerebral edema. Compared with the sham group, the ratio of the bilateral cerebral hemisphere volume markedly elevated on account of undergoing left MCAO surgical operation (Figure 3D). Similar to the result of Nim, treatment with QSYQ at three doses inhibited the ratio of the bilateral cerebral hemisphere volume, which confirmed that QSYQ treatment reduced cerebral edema in the subacute phase of the experimental animal model of ischemic stroke (Figure 3D).

### QSYQ Attenuated BBB Disruption in Subacute Stroke Rats

**FIGURE 4:**
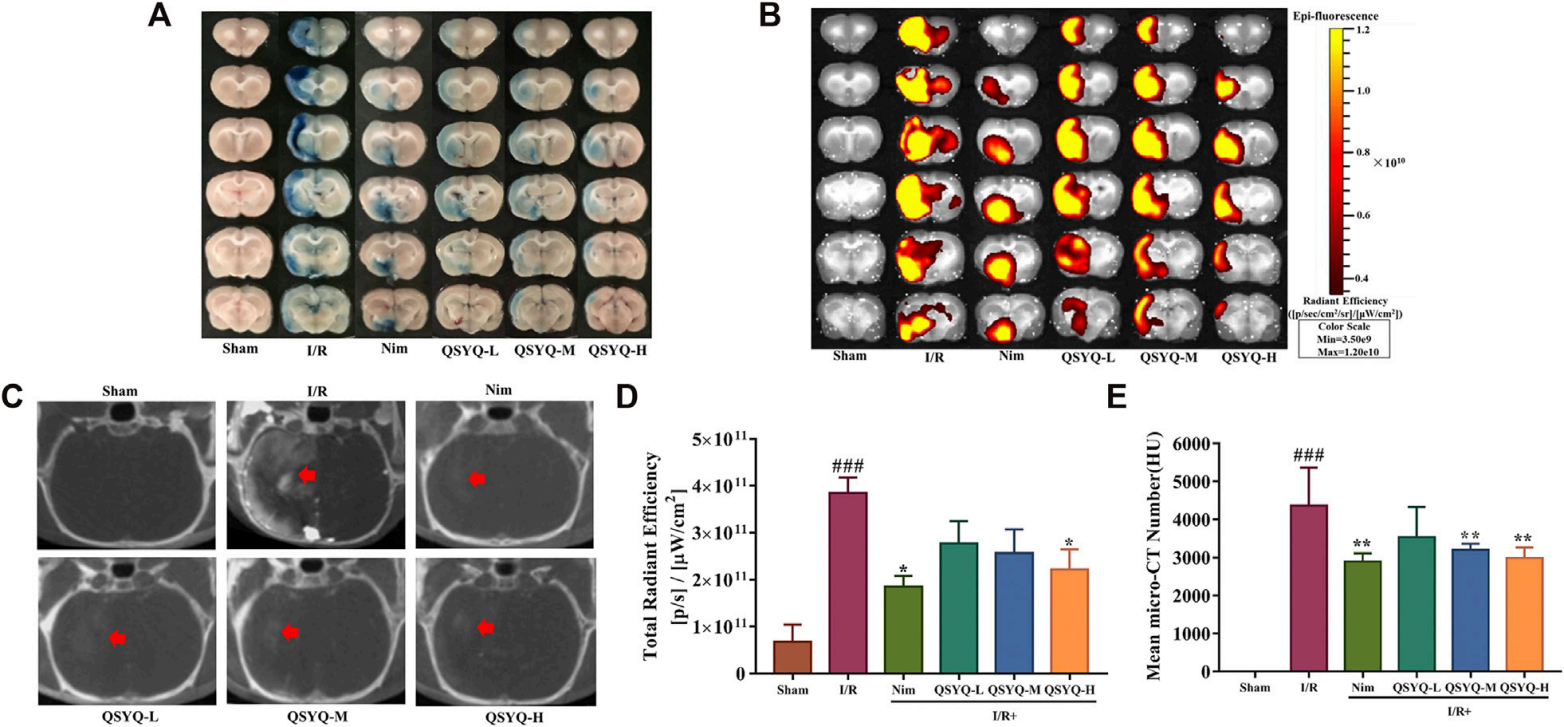
Treatment with QSYQ reduced the BBB breakdown in subacute stroke rats. **(A)** Representative images of cerebral coronal slices of the experimental rats stained with EB dye after undergoing ischemic stroke (n = 5–6). **(B)** The corresponding fluorescent pictures of EB leakage of brain coronal sections (n = 5–6). **(C)** Representative cerebral micro-CT images of Omnipaque extravasation of each group (n = 6). Red arrows indicate the position of Omnipaque extravasation. **(D)** Quantitative analysis of EB leakage of cerebral coronal sections of rats in each group. Treatment with QSYQ at high dose/Nim markedly reduce EB leakage caused by BBB disruption compared with the model group. **(E)** Quantitative analysis of brain Omnipaque leakage of rats from each group. Treatment with QSYQ at middle and high doses as well as Nim significantly lowered Omnipaque extravasation caused by BBB disruption. The data of total radiant efficiency are expressed as mean ± SEM. The data of Omnipaque leakage are presented as mean SD. #*p*< 0.05, ^**##**^ *p* < 0.01, and ^**###**^ *p* < 0.001 vs. the sham group; ^*^ *p*< 0.05, ^**^ *p*< 0.01, and ^***^ *p*< 0.001 vs. the model group. I/R, ischemia/reperfusion; Nim, Nimodipine; QSYQ-L, QSYQ 150°mg/kg-low dose; QSYQ-M, QSYQ 300°mg/kg-middle dose; QSYQ-H, QSYQ 600°mg/kg-high dose.

IVIS fluorescent imaging and brain micro-CT imaging techniques were integrated to detect and quantify the BBB integrity. As a control, no EB extravasation, Omnipaque leakage, and detectable spectrum were observed in the sham group, whereas a significant leakage (EB and Omnipaque) of the BBB was detected in the model samples (Figure 4). Notably, treatment with both Nim and QSYQ high dose (600 mg/kg) significantly lowered EB extravasation and Omnipaque leakage (Figure 4). Treatment with QSYQ at middle dose (300 mg/kg) also reduced Omnipaque leakage, whereas treatment with QSYQ at middle dose (300 mg/kg) attenuated EB leakage to some extent, but did not reach significance (Figures 4D,E). The results of EB and Omnipaque extravasation indicated that treatment with QSYQ at high dose (600 mg/kg) or Nim observably reduced BBB disruption of model rats in the subacute phase of experimental stroke.

### Identification of Differentially Expressed Proteins Induced by QSYQ Treatment in the Brain of Subacute Stroke Rats.

**FIGURE 5:**
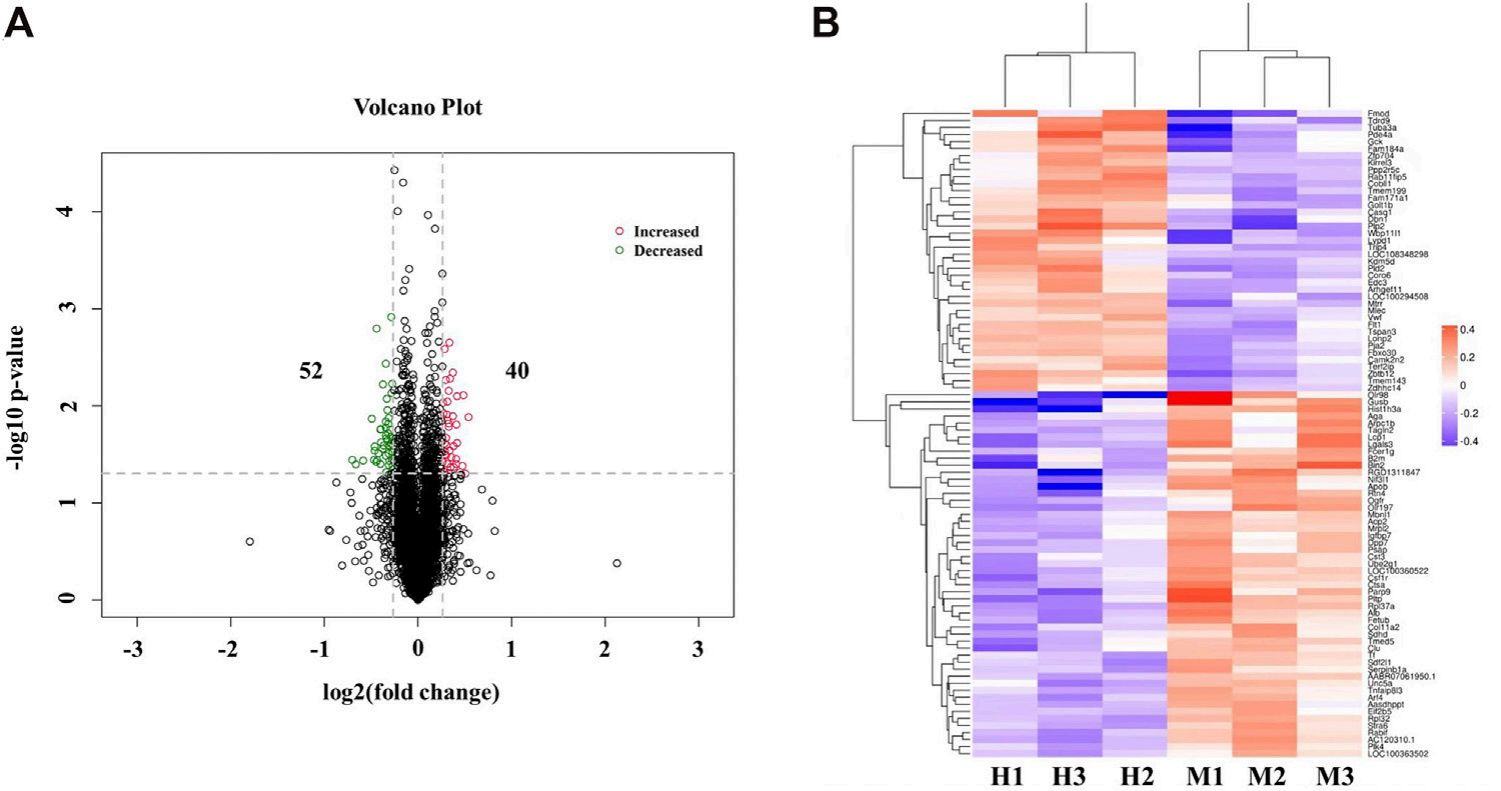
Identification of differentially expressed proteins between QSYQ-treated group and the model group in ischemic brain of subacute stroke rats. **(A)** Volcano plot of differentially expressed proteins regulated by QSYQ treatment. The differentially expressed proteins in tested samples were presented in different colors. Red stands for the proteins of increased expression, green stands for the proteins of decreased expression, and black stands for proteins that are not differently expressed. **(B)** A tree heat map of hierarchical clustering analysis of the individual brain samples (n = 3). X-coordinates represented samples and Y-coordinates represented the gene name of differentially expressed proteins. The log2 (fold change) value of differentially expressed proteins in tested samples was expressed in different colors in the heat map, with red indicating up-regulation and blue indicating down-regulation. H means QSYQ at high dose (600 mg/kg) group; M means model group.

To reveal the underlying molecular mechanisms of subacute phase (1.5 h ischemia and 8 days reperfusion) stroke protection against cerebral injury by QSYQ, TMT-based quantitative proteomics analysis was employed on the proteins extracted from ischemic brain tissues of MCAO/reperfusion rats with or without QSYQ treatment. A total of 34,187 unique peptides were detected (only rank 1 peptides and FDR ≤1% were accepted) and 5,813 proteins were identified by one or more unique peptides. Using a strict criteria (fold change >1.20 or <0.833, *p*-value <0.05), a total of 92 differentially expressed proteins in ischemic brain tissues were detected between QSYQ (high dose)-treated and the model groups. Among them, the expression levels of 40 proteins were significantly elevated with a fold change more than 1.20 in H (QSYQ high dose group) vs. M (model group), whereas the expression levels of 52 proteins were dramatically reduced with a fold change less than 0.833 in H (QSYQ high dose group) vs. M (model group), as displayed in a volcano plot in Figure 5A. Hierarchical clustering analysis of the acquired protein relative expression data were visualized in a tree heat map (Figure 5B), which supported the rationality of selecting differentially expressed proteins. The detailed information of significantly differentially expressed proteins was shown in Supplementary Table S2.

### Network Pharmacology Analysis Revealed Galectin-3 Mediated Neuroinflammation as a Sey Mechanism of Subacute Stroke Protection by QSYQ.

**FIGURE 6:**
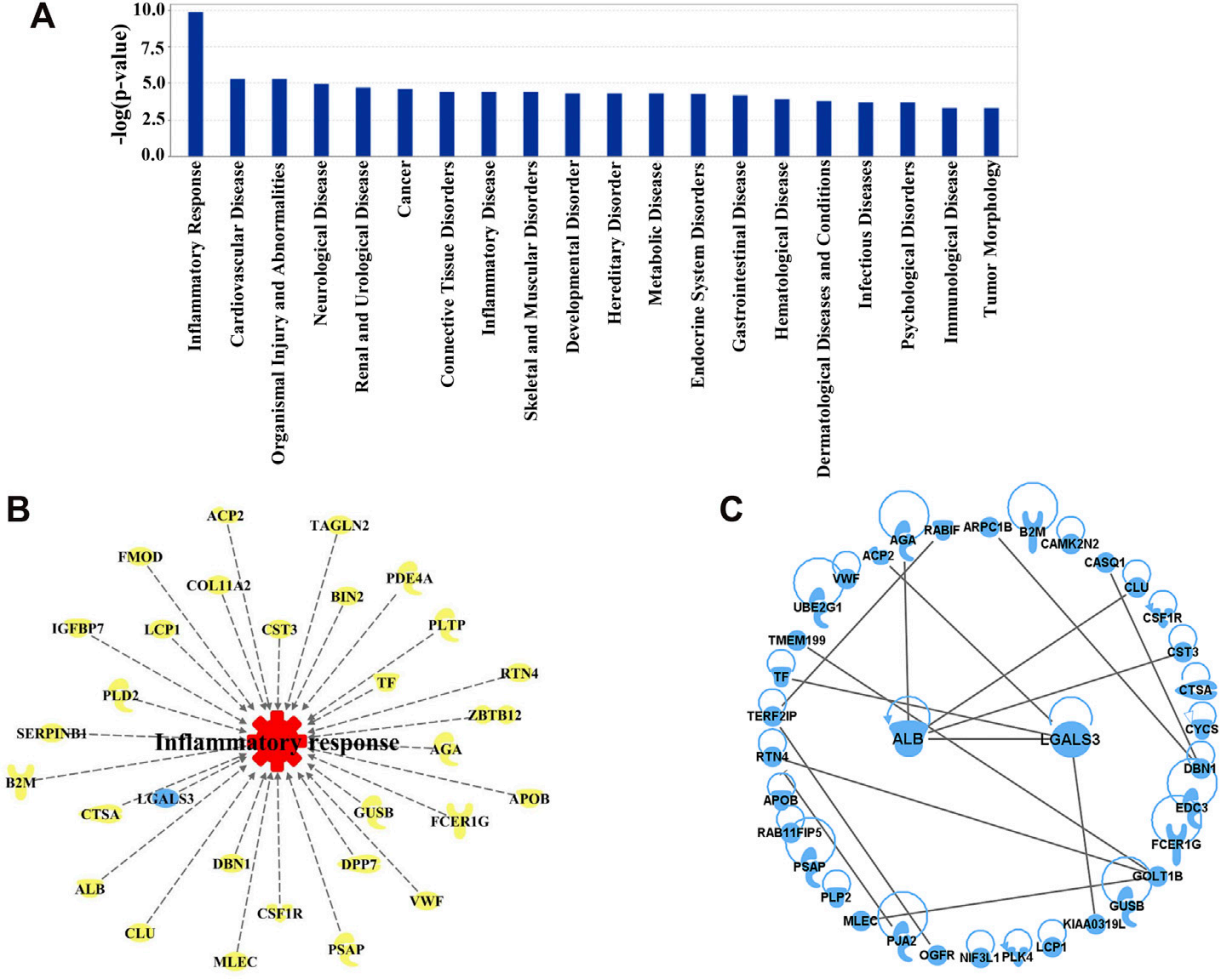
**(A)** Diseases and Functions affected by QSYQ were ranked according to the Fisher’s exact test algorithm. Top 20 diseases and functions were displayed in descending order based on -log (*p*-value) score. Among them, inflammatory response ranked the highest with a -log (*p*-value) score. **(B)** 30 important proteins correlated with inflammatory response picked out from 92 differentially expressed proteins modulated by QSYQ. **(C)** PPI network of the differentially expressed proteins. From the network, galectin-3 (LGALS3) and albumin (ALB) directly interact with the greatest number of proteins.

To discover the molecular mechanisms accounting for the action of QSYQ treatment against ischemia and reperfusion injury, network pharmacology analysis was carried out using IPA system. The “Core analysis-Diseases and Functions” module was executed to gain the top diseases and functions strongly linked to the significantly differentially expressed proteins. Based on the Fisher’s exact test algorithm of IPA, the diseases as well as functions were ranked according to corresponding *p*-value scores so as to distinguish the relatedness or importance among these proteins and diverse diseases as well as functions. The top 10 diseases and functions influenced by QSYQ in the order of descending **-**log (*p*-value) score were inflammatory response, cardiovascular disease, organismal injury and abnormalities, neurological disease, renal and urological disease, cancer, connective tissue disorders, inflammatory disease, skeletal and muscular disorders, and developmental disorder. As the inflammatory response ranked the highest with a **-**log (*p*-value) score (Figure 6A), the result indicated that the anti-inflammatory mechanism could be one of the most vital mechanisms to explain QSYQ effective action against experimental stroke. The corresponding differentially expressed proteins involved in inflammatory response determined by “Build-Grow-Diseases and Functions” module of IPA (Figure 6B) showed that 30 proteins, including galectin-3 (LGALS3), albumin (ALB), cathepsin A (CTSA), clusterin (CLU), and transferrin (TF), participated in the inflammatory response. PPI network analysis positioned galectin-3 (LGALS3) and albumin (ALB) as the core proteins regulated by QSYQ since they displayed the most interactions with other significantly differentially expressed proteins revealed by our differential proteomic analysis (Figure 6C). The detailed information of PPI network analysis was shown in Supplementary Table S3.

### QSYQ Reversed Galectin-3 Mediated Neuroinflammation in Subacute Stroke Brain Tissues

**FIGURE 7:**
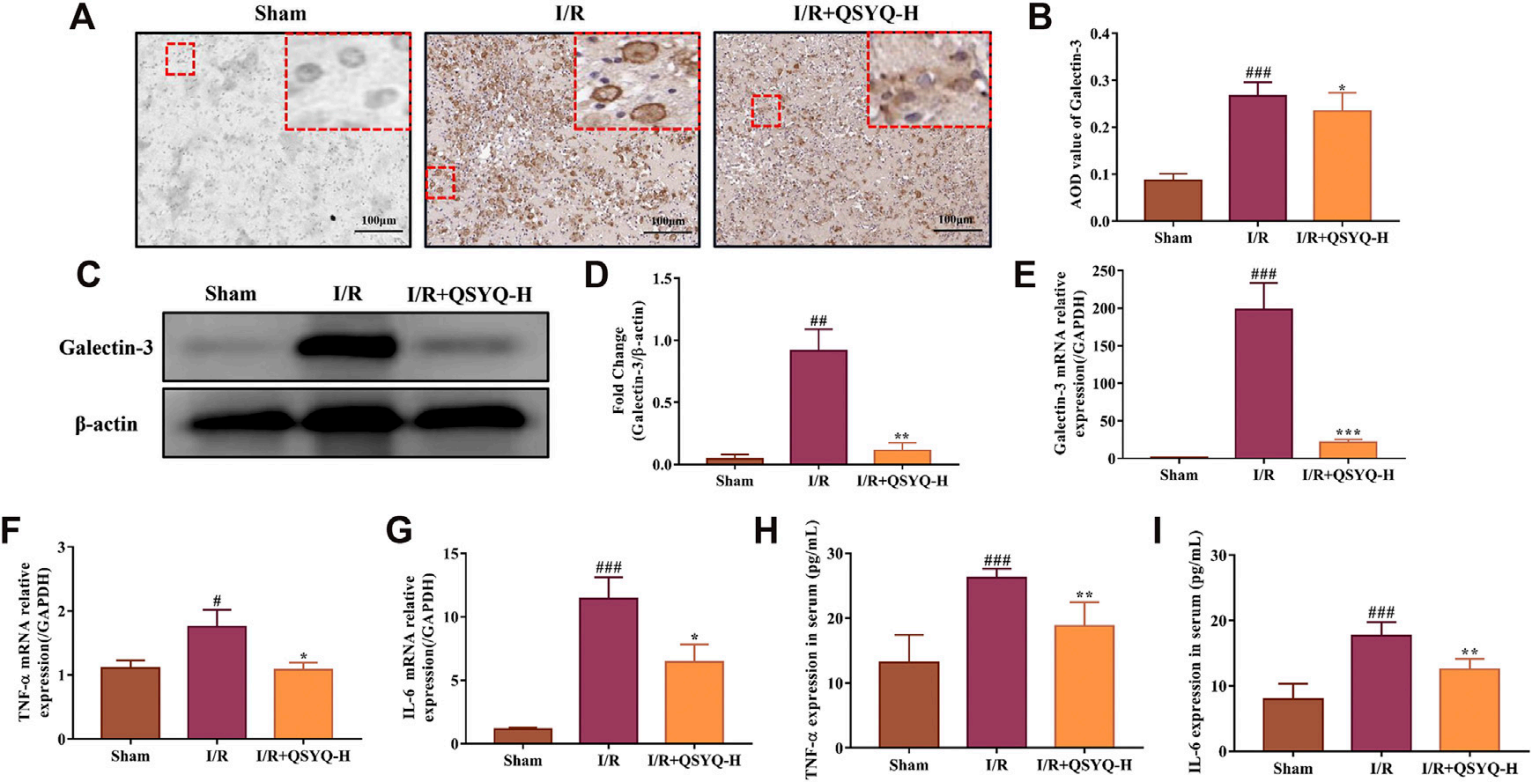
The regulatory role of QSYQ on galectin-3 mediated neuroinflammation in response to cerebral ischemia and reperfusion injury. **(A)** Representative images of immunohistochemical staining of galectin-3 protein expression in brain tissue slices (×200 magnification). **(B)** Quantification of immunohistochemical staining (n = 3). **(C)** Representative images of western blotting of galectin-3. **(D)** The quantitative analysis of western blotting (n = 3). Treatment with high doses of QSYQ significantly reduced the protein expression level of galectin-3 compared with the model group. Relative expression levels of cerebral LGALS3 **(E)**, TNF-α **(F)**, and IL-6 **(G)** mRNA were evaluated by RT-qPCR (n = 3). Results were normalized to GAPDH expression. Treatment with high dose of QSYQ significantly decreased the mRNA expression of galectin-3-mediated inflammation related genes. Quantitative assessment the effect of QSYQ treatment on the release of TNF-α **(H)** and IL-6 **(I)** in rats serum following MCAO and reperfusion using ELISA kits (n = 6). The data of western blotting and ELISA are expressed as mean ± SD. Meanwhile, the data of RT-qPCR are presented as mean ^**#**^ *p* < 0.05, ^**##**^ *p* < 0.01, and ^**###**^ *p* < 0.001 vs. the sham group; ^*^ *p*< 0.05, ^**^ *p*< 0.01, and ^***^ *p*< 0.001 vs. the model group. I/R, ischemia/reperfusion; QSYQ-H, QSYQ 600°mg/kg-high dose.

Based on the results of network pharmacology analysis, the differentially expressed protein of galectin-3 was found to act as a critical target involved in QSYQ-regulated inflammatory response during subacute stroke recovery. Both immunohistochemistry and western blotting were performed to confirm the results from the TMT-based quantitative proteomics analysis. As shown in Figures 7A–D, the protein expression level of galectin-3 was significantly increased in brain tissues of stroke rats in the subacute phase compared with the sham group. Treatment with high dose (600 mg/kg) of QSYQ markedly decreased galectin-3 expression compared to rats that underwent MCAO and reperfusion (Figures 7A–D). This was consistent with the above quantitative proteomic datasets. Since published literature reported that galectin-3 activated TLR-4 and downstream inflammatory targets like nuclear factor κB (NF-κB), resulting in an increase in the release of pro-inflammatory cytokines following brain injury (Burguillos et al., 2015), we compared the mRNA expression of the following genes - LGALS3 (galectin-3), TNF-α, and IL-6 - in rat brain samples from the sham group, cerebral I/R injury model group, and model treated with QSYQ (600 mg/kg) group by RT-qPCR. Compared with the sham group, MCAO and reperfusion caused obvious inflammation, as indicated by significant upregulation of these mRNA levels (Figures 7E–G). Importantly, treatment with high dose (600 mg/kg) of QSYQ distinctly downregulated mRNA expression of these neuroinflammation-related genes compared to the model group (Figures 7E–G).

### QSYQ Reduced Proinflammatory Factors TNF-α and IL-6 in Serum

To gain an insight into the regulatory role of QSYQ on galectin-3 mediated inflammation in subacute ischemic stroke, serum levels of TNF-α and IL-6 in different groups were determined by ELISA. As shown in Figures 7H,I, concentrations of TNF-α and IL-6 in serum were significantly higher in rats that underwent MCAO/reperfusion compared with that from sham-operated rats. As expected, treatment with QSYQ at high dose (600 mg/kg) markedly lowered the serum concentrations of TNF-α and IL-6 compared with the model group (Figures 7H,I).

## Discussion

Recovery after stroke is a complex, dynamic, multifactorial, and multicellular process (Alawieh et al., 2018; Sadler et al., 2020). No drugs or therapies have been approved for stroke recovery to date, in part due to inappropriate experimental models and because screen strategies focusing on single targets and drug molecules were used (Cramer, 2015). Our current study tested a hypothesis that a multi-component medicine, such as QSYQ, targeting different biological pathways would be more effective for post-stroke recovery. At present, QSYQ with the function of Qi-tonifying and blood-activating has been widely used to treat cardiovascular disease in clinic (Han et al., 2019). In addition to the cardioprotection against ischemia/reperfusion injury, we recently also reported the protective effect of pretreatment with QSYQ against CI/RI, which may be partly related to the modulation of neuroinflammatory response in an acute stroke model (Chen et al., 2015; Wang et al., 2020). However, questions remain on whether QSYQ contributes to the therapeutic post-stroke recovery and, if so, what the underlying mechanisms are.

To evaluate the efficacy of QSYQ for post-stroke recovery, we chose Nim as a positive control drug in the present study. Nim is a dihydropyridine calcium channel blocker and has been observed for its therapeutic application in stroke. Babu et al. showed that administration of Nim alleviated excitotoxicity and neurological deficits in experimental ischemic stroke rats (Babu and Ramanathan, 2011). Liu et al. found that Nim treatment reduced infarct volume and pathological damage and improved neurological function of subacute stroke animals (Liu et al., 2016). The beneficial effect of Nim on protecting BBB integrity has also been reported (Li et al., 2019). Since neurological and motor deficits are common in stroke patients, improvement of neural and behavioral outcomes are considered as necessary to estimate the action of anti-stroke drugs (Benjamin et al., 2019). Therefore, in addition to the parameters used in our previous acute CI/RI study, mNSS, open field test, and gait test were integrated to systematically evaluate the neuroprotective function of QSYQ on promoting post-stroke recovery in this work. Similar to Nim, the results indicated that treatment with QSYQ at middle (300 mg/kg) and high dose (600 mg/kg) markedly inhibited cerebral infarct volume as well as ameliorated neurological and behavioral impairment in subacute stroke rats (Figures 1–3), which revealed the beneficial effect of QSYQ on functional recovery.

BBB dysfunction, characterized by the structural disruption of tight junction protein complexes causing increased permeability, ion transporter dysfunction leading to cerebral edema, and further inflammatory damage induced by the infiltration and accumulation of peripheral immune cells and the activation of resident microglial cells, is a prominent pathological feature of ischemic stroke (Jiang et al., 2018). Damage to BBB permeability after cerebral ischemia and reperfusion is apparent in both the acute and subacute phases of ischemic stroke (∼1–7°days) (Jiang et al., 2012). In response to CI/RI, multiple mechanisms may be involved in the restoration of BBB integrity, such as inflammatory response, oxidative stress, and angiogenesis (Jiang et al., 2018). The therapeutic strategies to promote BBB repair may enhance the functional recovery of the subject after ischemic stroke (Boese et al., 2018). In this study, the IVIS fluorescent imaging and brain micro-CT imaging techniques were respectively applied to measure BBB permeability. Significantly, treatment with QSYQ at high dose (600 mg/kg) and Nim obviously reduced the extravasation of EB and Omnipaque on account of BBB breakdown after ischemic stroke, which suggested the positive action of QSYQ against BBB disruption caused by ischemia and reperfusion (Figure 4). The data of micro-CT imaging also indicated decreased cerebral edema after three doses of QSYQ treatment, which could be observed in the subacute stage of CI/RI (Figures 3C,D).

Proteomics analysis takes a holistic approach to deciphering the complexity of biological systems and to characterizing protein interactions and biological networks that mediate physiological and pathological processes of diseases (Ji et al., 2015). In recent years, it has been increasingly applied to reveal the underlying mechanisms of TCM for multiple therapeutic effects on stroke treatment (Ning et al., 2012; Ji et al., 2015). TMT-based proteomics technique, employing chemical peptide labeling with amine-reactive isobaric tags, is a reliable method for large-scale multiplexed proteins relative quantification between multiple samples within a single experiment (Rauniyar et al., 2013). In this study, we applied TMT-based proteomics (TMT-LC-MS/MS) to label ischemic rat brain tissue samples to identify differentially expressed proteins following QSYQ treatment. As a result, a total of 5,813 proteins were identified, of which 92 were significantly differentially expressed (fold change >1.20 or <0.833, *p*-value <0.05) (Figure 5A). IPA analysis revealed that among the differentially expressed proteins, those with the highest -log (*p*-value) score were closely associated with inflammatory response, suggesting that post-stroke inflammation may be one of the main mechanisms underlying the beneficial action of QSYQ in enhancing functional recovery after CI/RI (Figures 6A,B). Importantly, subsequent PPI network analysis revealed that an inflammation-related protein named galectin-3 (LGALS3) could be tightly networked with other proteins (Figure 6C) and serve as a key player of QSYQ action at the subacute phase of stroke.

Inflammation has been recognized as a double-edged sword which could be either detrimental or beneficial depending on the particular stages after a stroke. In response to acute ischemic stroke, resident glial cells activation, infiltration of peripheral immune cells, release of pro-inflammatory cytokines and BBB disruption together orchestrate the post-stroke augmented inflammatory response, which contribute to a series of long-term secondary brain damages and neurological behavioral deficits (Rajkovic et al., 2018; Wang et al., 2018). In the late repair phase of CI/RI, neuroinflammation fundamentally participates in multiple brain recovery processes, including post-stroke neurogenesis, neurovascular unit remodeling, post-stroke synaptogenesis, and axonal sprouting, which synergistically promote neurological behavioral outcomes (Wang et al., 2018). Currently, the regulation of inflammation is considered as a primary target for the development of stroke therapies.

Recently, growing evidence has suggested that galectin-3 could act as an endogenous regulator of brain injury-related inflammation (Shin, 2013; Rahimian et al., 2018). In response to cerebral damage induced by stroke, high level expression of galectin-3 was observed in activated microglial cells localized in the ischemic lesion (Lalancette-Hebert et al., 2012). Burguillos *et al.* showed that microglia-secreted galectin-3 acted as an endogenous ligand for TLR-4 activation to amplify the pro-inflammatory response and led to hippocampal degeneration in the injured brain caused by ischemia (Burguillos et al., 2015). However, galectin-3 deficiency reduced the expression of pro-inflammatory cytokines and exerted a neuroprotective effect (Yip et al., 2017). Prins *et al.* reported that the absence of galectin-3 attenuated neuroinflammation and ameliorated neurological functional recovery after spinal cord injury (Prins et al., 2016). These findings suggested that galectin-3 could be regarded as a major immunomodulatory molecule that played a significant role in the modulation of brain inflammation and neurodegeneration. In this study, we found that treatment with QSYQ (600 mg/kg) distinctly downregulated the high expression level of galectin-3 protein in brain tissues from MCAO/reperfusion rats according to the quantitative proteomics analysis results, which was verified with the methods of RT-qPCR, immunohistochemistry, and western blot analysis (Figures 7A–E). The results indicated that the neuroprotective function of QSYQ against the subacute phase of CI/RI might act partly through the regulation of galectin-3-mediated inflammatory response.

The inflammatory cytokines, tumor necrosis factor-alpha (TNF-α) and interleukin-6 (IL-6), are generally considered to be representative contributors to neuroinflammation in ischemic stroke and are therefore promising potential targets in future stroke therapy (Lambertsen et al., 2019). In experimental stroke model of rodents, the increased expression levels of TNF-α mRNA and protein were observed in ischemic brain tissues (Berti et al., 2002; Haddad et al., 2006). In the subacute phase of CI/RI, the upregulated level of TNF-α and IL-6 contents could be detected in damaged brain tissues (Yang et al., 2019). The enhanced serum levels of TNF and IL-6 in stroke patients were tested on days 1, 3, and 7 after stroke when compared to the control group (Jiang et al., 2017). Furthermore, TNF-α was also reported to regulate neuronal networks involved in cognition and behavior (Baune et al., 2008). In the present work, we further showed that treatment with QSYQ (600 mg/kg) significantly reduced the mRNA levels of TNF-α and IL-6 in brain tissues of stroke rats after ischemia and reperfusion (Figures 7F,G). Moreover, the serum levels of TNF-α and IL-6 were markedly increased in the subacute phase of stroke, which were reversed by QSYQ (600 mg/kg) (Figures 7H,I). The data suggested that the beneficial effects of QSYQ on brain damage and neurological behavioral dysfunction induced by experimental stroke were partly *via* the modulation of galectin-3-mediated inflammatory reaction.

## Limitation and Future Directions

Although we demonstrated the efficacy and mechanism of QSYQ for post-stroke recovery, there are still limitations to this study. First, the clinical relevance of the rat subacute stroke model we used needs to be tested in human studies. Secondly, models of longer-term recovery are to be established and optimized to evaluate drugs and treatments that will benefit real-time patients. Thirdly, differential contributions of Yiqi (Qi-benefitting, i.e., Huangqi) and Huoxue (Blood-activiting, i.e., Danshen + Sanqi) components of QSYQ need to be distinguished and the active components involved needs defining.

## Conclusion

In summary, the current work provides the first evidence for the beneficial action of treatment with QSYQ against the subacute phase of CI/RI, which may be closely associated with its potential to inhibit MCAO/reperfusion-induced upregulation of brain-injury-related inflammatory regulator galectin-3, as well as the pro-inflammatory cytokines TNF-α and IL-6. The results suggest that QSYQ exerts a neuroprotective role in the damaged brain caused by experimental stroke probably through modulating the galectin-3-mediated inflammatory response. The fact that QSYQ can improve post-stroke outcome needs further clarification, and more thorough and reliable experimental data are required to support its clinical application. Our study may provide a novel therapeutic agent of ischemic stroke.

## Data Availability

All datasets generated for this study are included in the article and the Supplementary Material. Besides, the mass spectrometry proteomics data were deposited to the ProteomeXchange via the PRIDE partner repository with the dataset identifier PXD020936.
