# Supplementary material for: Galectin-3 Mediated Inflammatory Response Contributes to Neurological Recovery by QiShenYiQi in Subacute Stroke Model
Source: Front Pharmacol. 2021 Apr 19;12:588587. doi: 10.3389/fphar.2021.588587 (PMC8089377; doi:10.3389/fphar.2021.588587)
Supplement: Supplementary file 1 [file datasheet1.zip › Supplemental Table S1 Modified Neurological Severity Score.docx]

**Supplemental Table S1** Modified Neurological Severity Score (mNSS)

| **Motor Tests** | 6 |
| --- | --- |
| Raising rat by the tail | 3 |
| Flexion of forelimb | 1 |
| Flexion of hindlimb | 1 |
| Head moved > 10° to vertical axis within 30 s | 1 |
| Placing rat on the floor (normal = 0; maximum = 3) | 3 |
| Normal walk | 0 |
| Inability to walk straight | 1 |
| Circling toward paretic side | 2 |
| Falls down to the paretic side | 3 |
| **Sensory tests** | 2 |
| Placing test (visual and tactile test) | 1 |
| Proprioceptive test (deep sensation, pushing the paw against table edge to stimulate limb muscles) | 1 |
| **Beam balance tests (normal = 0; maximum = 6)** | 6 |
| Balances with steady posture | 0 |
| Grasps side of beam | 1 |
| Hugs the beam and one limb falls down from the beam | 2 |
| Hugs the beam and two limbs fall down from the beam, or spins on beam( >60 s) | 3 |
| Attempts to balance on the beam but falls off (>40 s) | 4 |
| Attempts to balance on the beam but falls off (>20 s) | 5 |
| Falls off: no attempt to balance or hang on to the beam (<20 s) | 6 |
| **Reflexes absent and abnormal movements** | 4 |
| Pinna reflex (head shake when touching the auditory meatus) | 1 |
| Corneal reflex (eye blink when lightly touching the cornea with cotton) | 1 |
| Startle reflex (motor response to a brief noise from snapping a clipboard paper) | 1 |
| Seizures, myoclonus, myodystony | 1 |
| **Maximum points** | 18 |

One point is awarded for the inability to perform the tasks or for the lack of a tested reflex; 13-18 indicates severe injury; 7-12 indicates moderate injury; 1-6 indicates mild injury*.*
