# Supplementary figures and images for: Galectin-3 Mediated Inflammatory Response Contributes to Neurological Recovery by QiShenYiQi in Subacute Stroke Model
Source: Front Pharmacol. 2021 Apr 19;12:588587. doi: 10.3389/fphar.2021.588587 (PMC8089377; doi:10.3389/fphar.2021.588587)

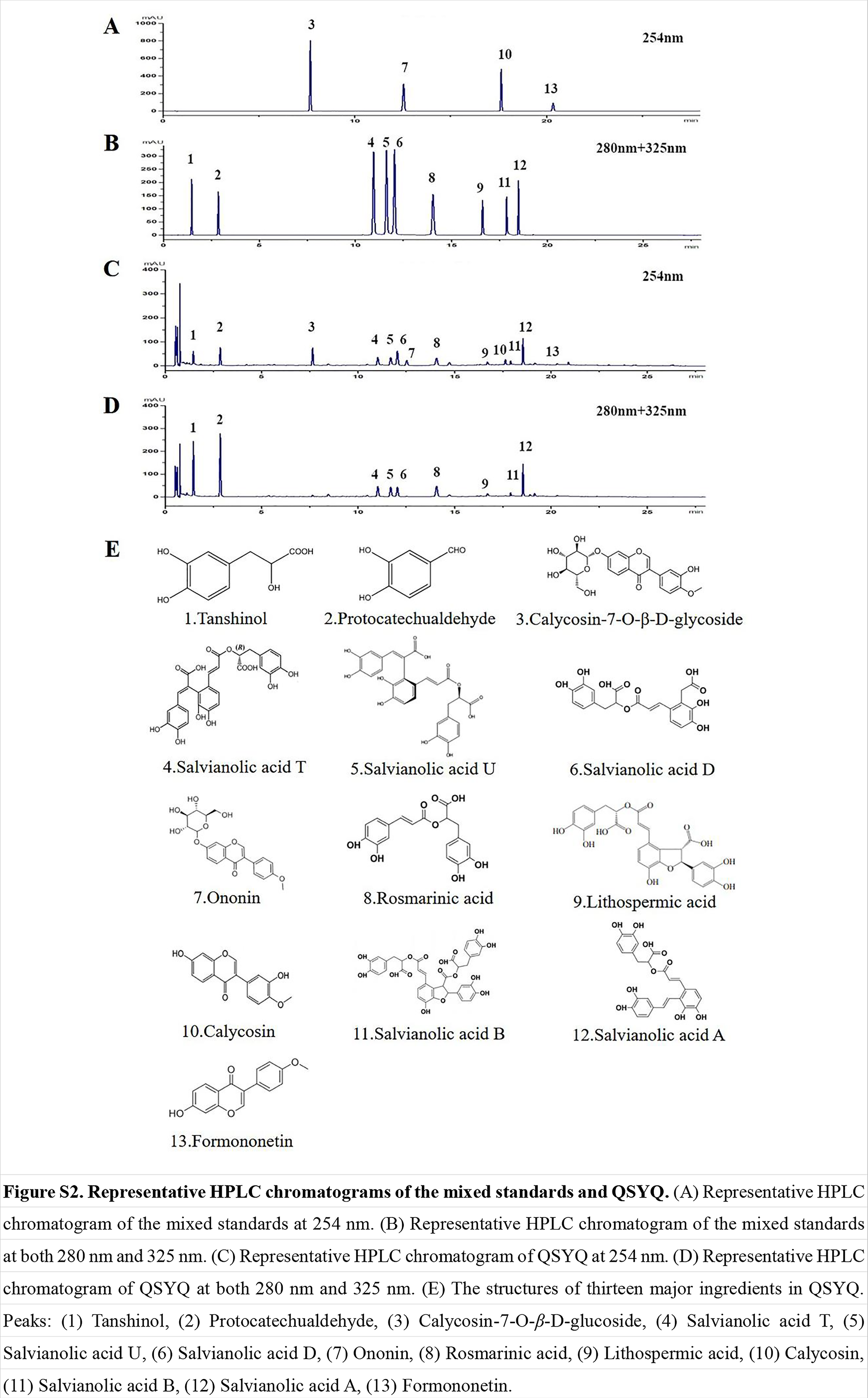

Supplement: Supplementary file 1 [file datasheet1.zip › Supplemental Figure S2.tif]

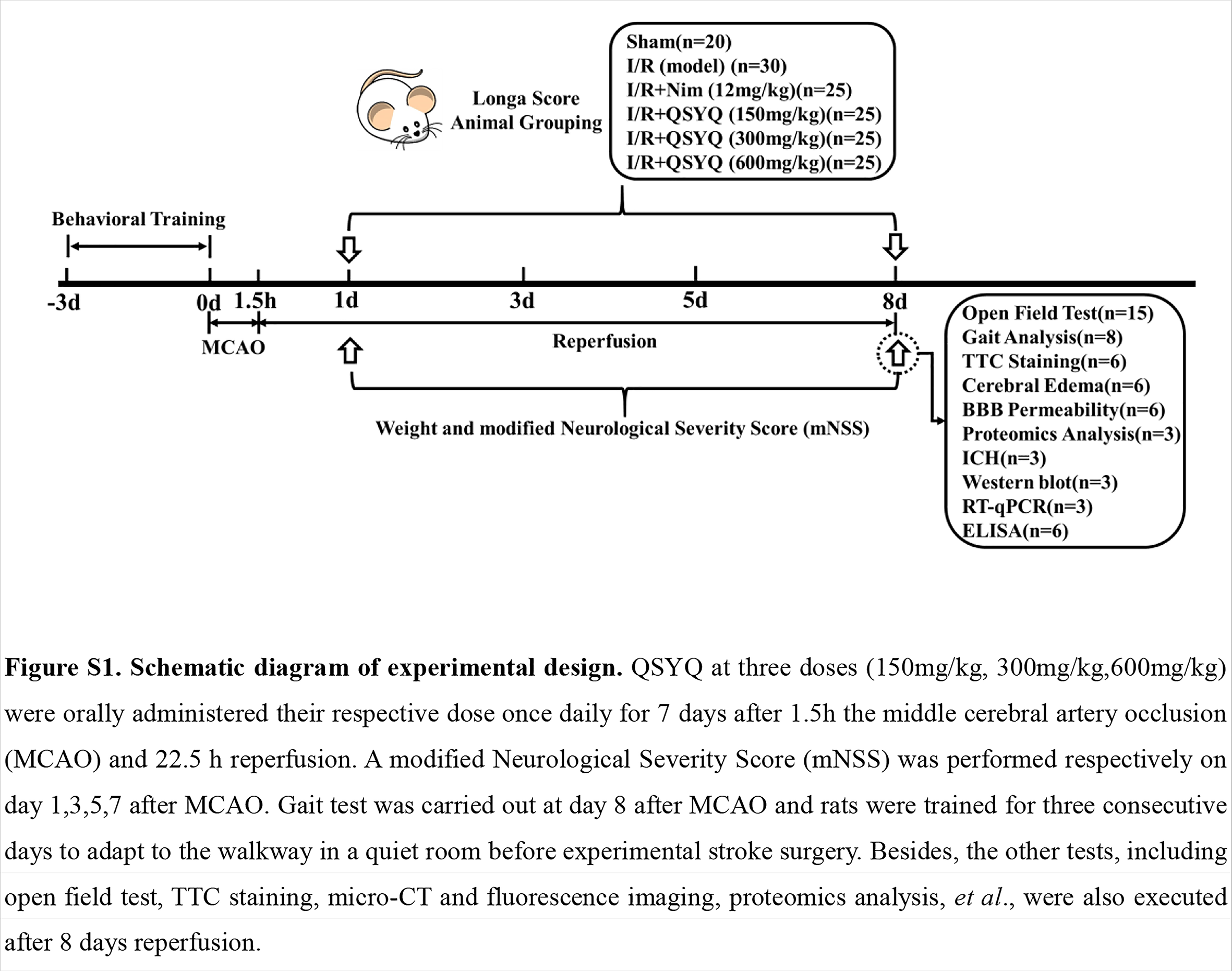

Supplement: Supplementary file 1 [file datasheet1.zip › Supplemental Figure S1.tif]

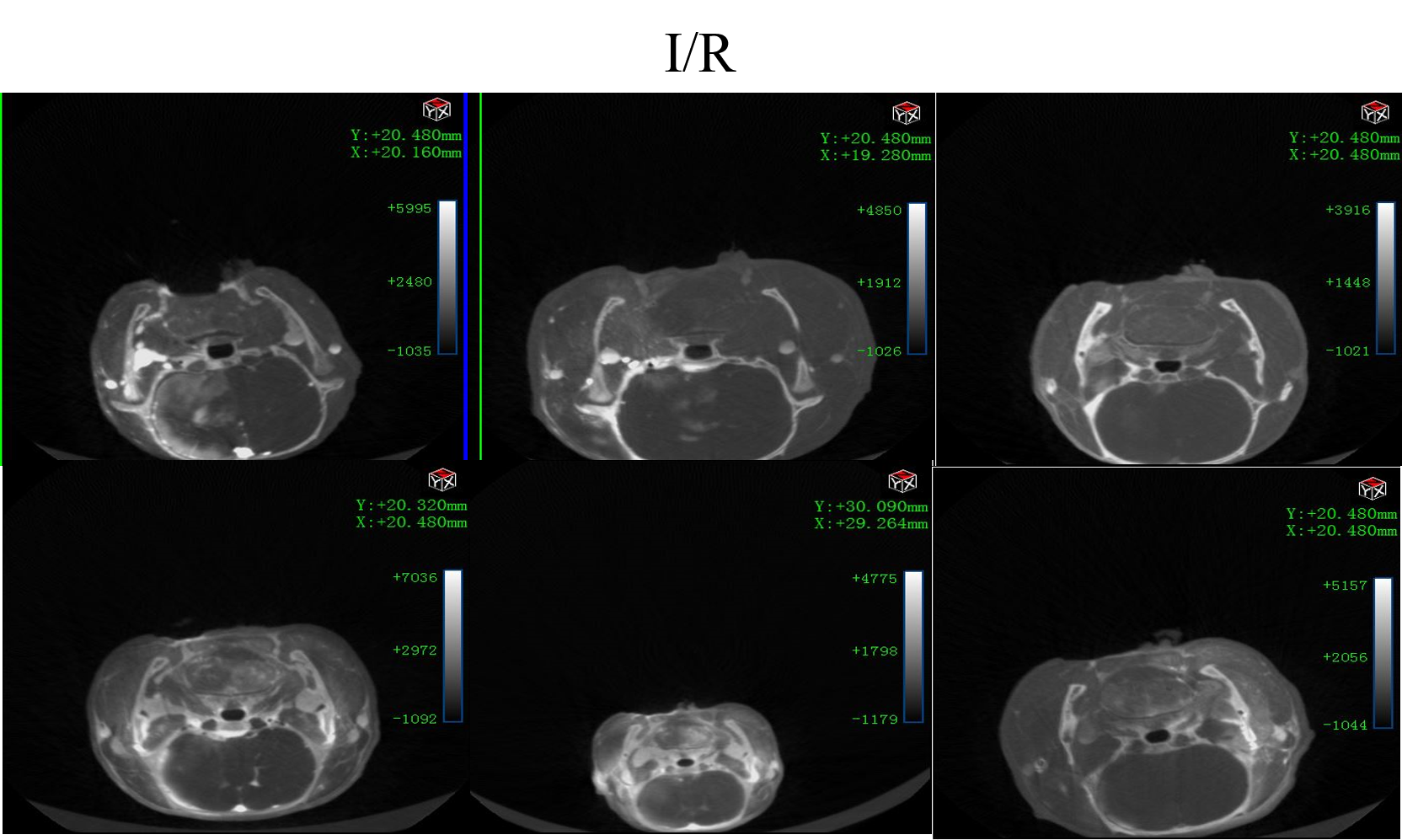

Supplement: Supplementary file 2 [file datasheet2.zip › CT-BBB-Model.tif]

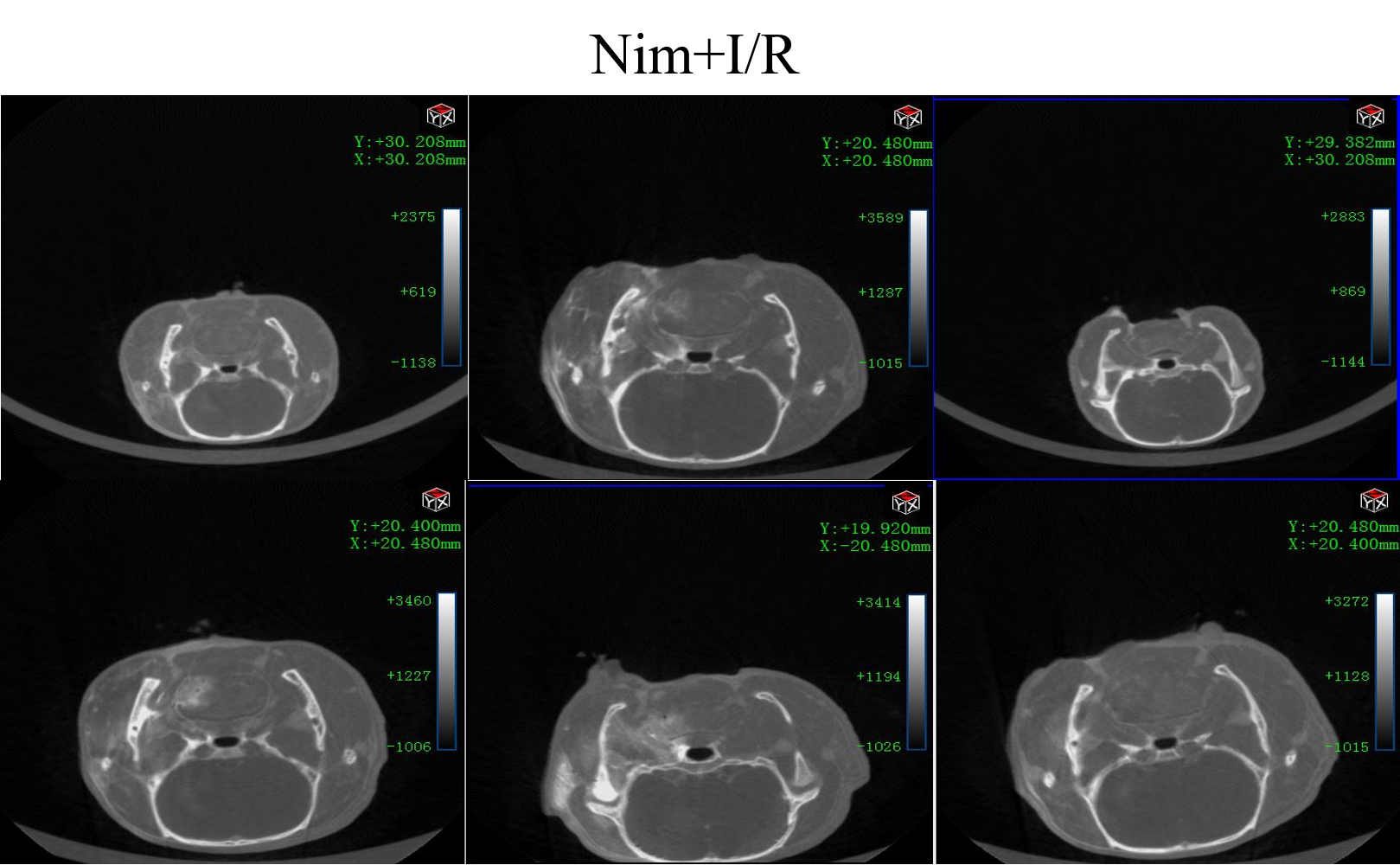

Supplement: Supplementary file 2 [file datasheet2.zip › CT-BBB-Nim.tif]

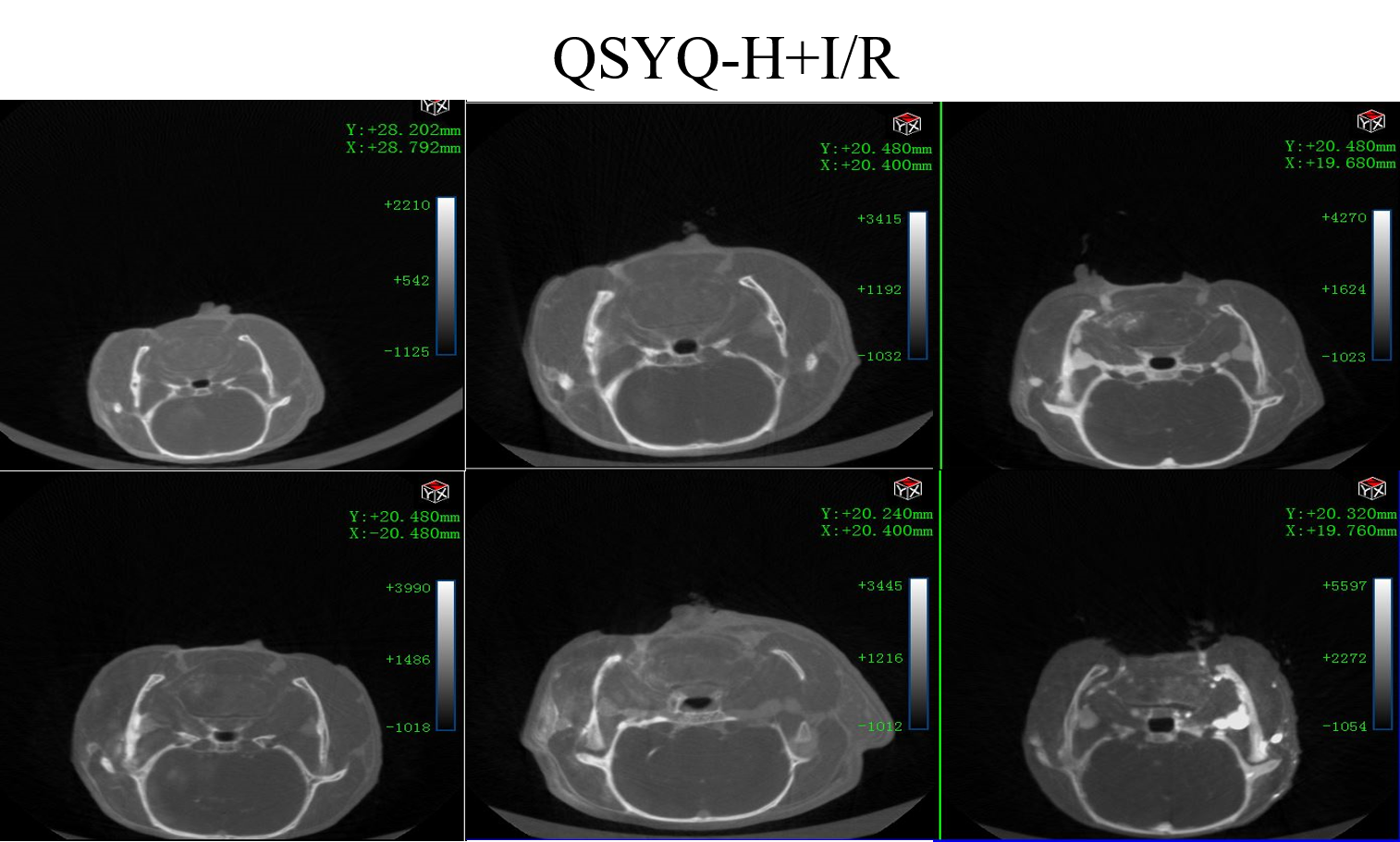

Supplement: Supplementary file 2 [file datasheet2.zip › CT-BBB-QSYQ-H.tif]

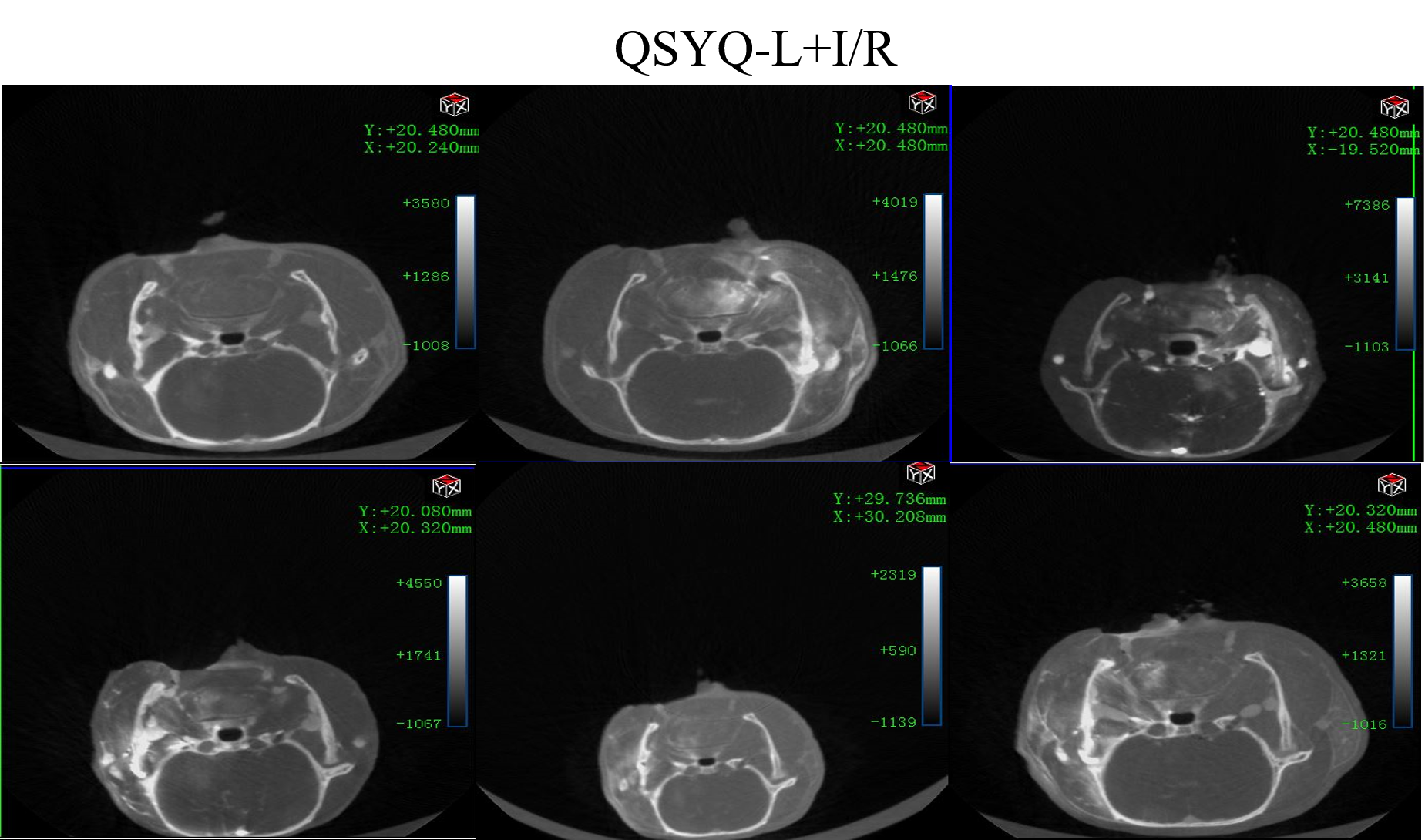

Supplement: Supplementary file 2 [file datasheet2.zip › CT-BBB-QSYQ-L.tif]

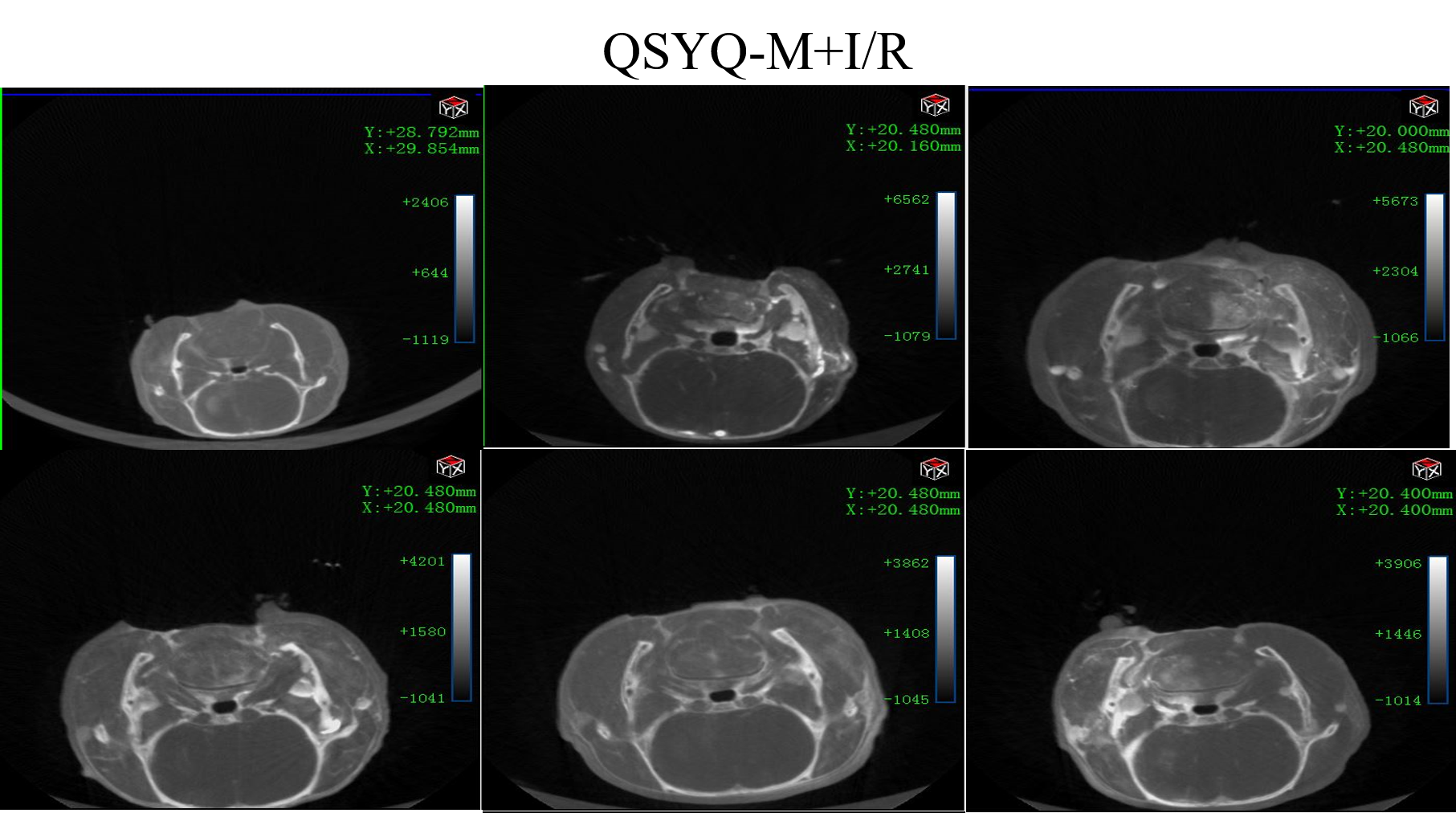

Supplement: Supplementary file 2 [file datasheet2.zip › CT-BBB-QSYQ-M.tif]

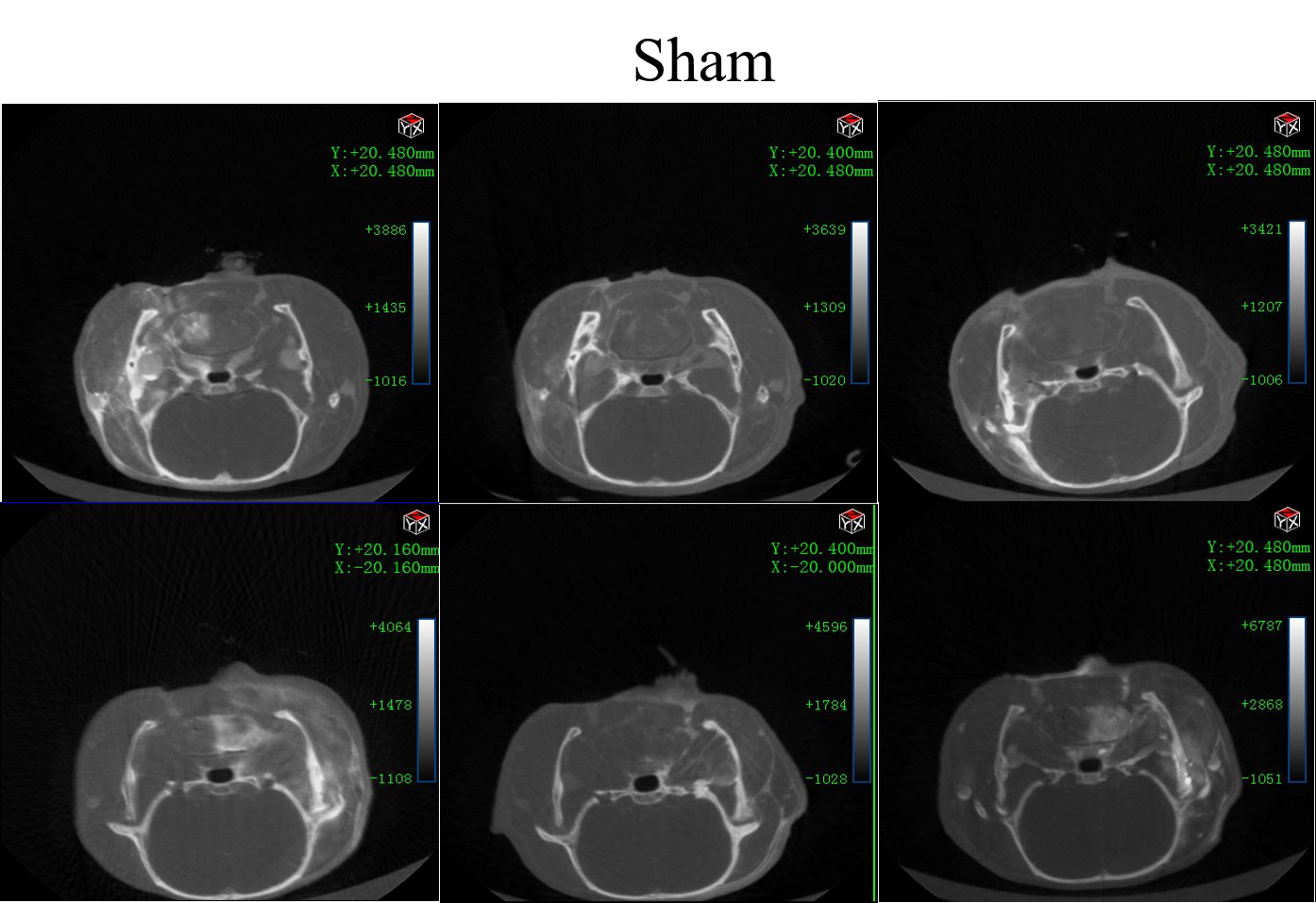

Supplement: Supplementary file 2 [file datasheet2.zip › CT-BBB-Sham.tif]

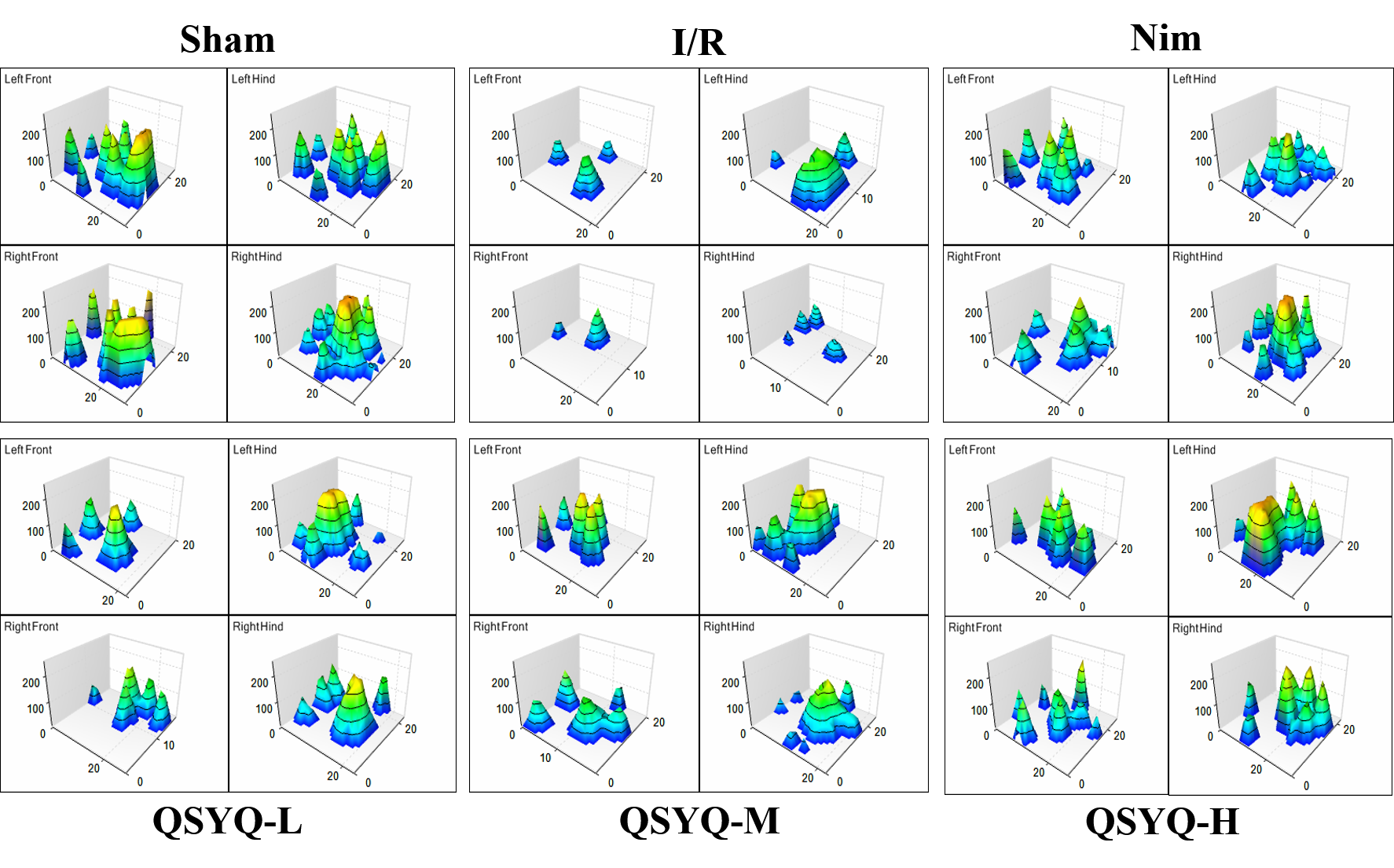

Supplement: Supplementary file 2 [file datasheet2.zip › CatWalk-3D.tif]

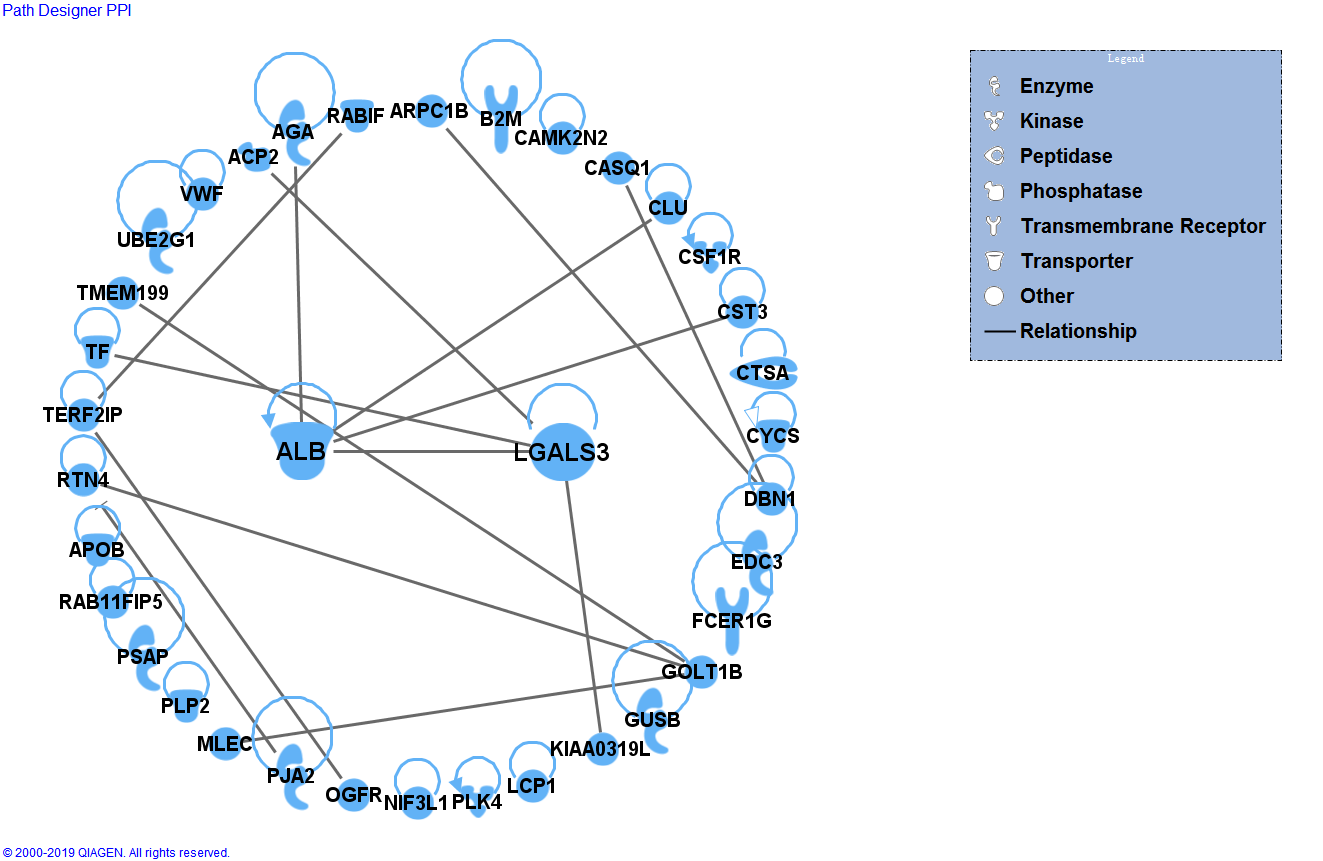

Supplement: Supplementary file 2 [file datasheet2.zip › IPA-PPI.png]

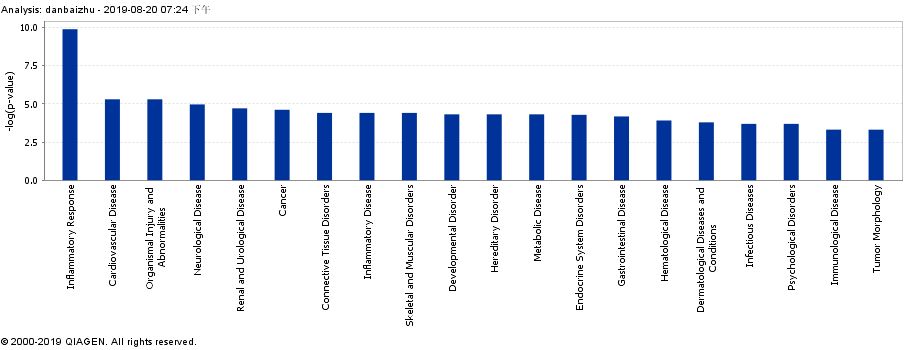

Supplement: Supplementary file 2 [file datasheet2.zip › IPA-diseases and functions.png]

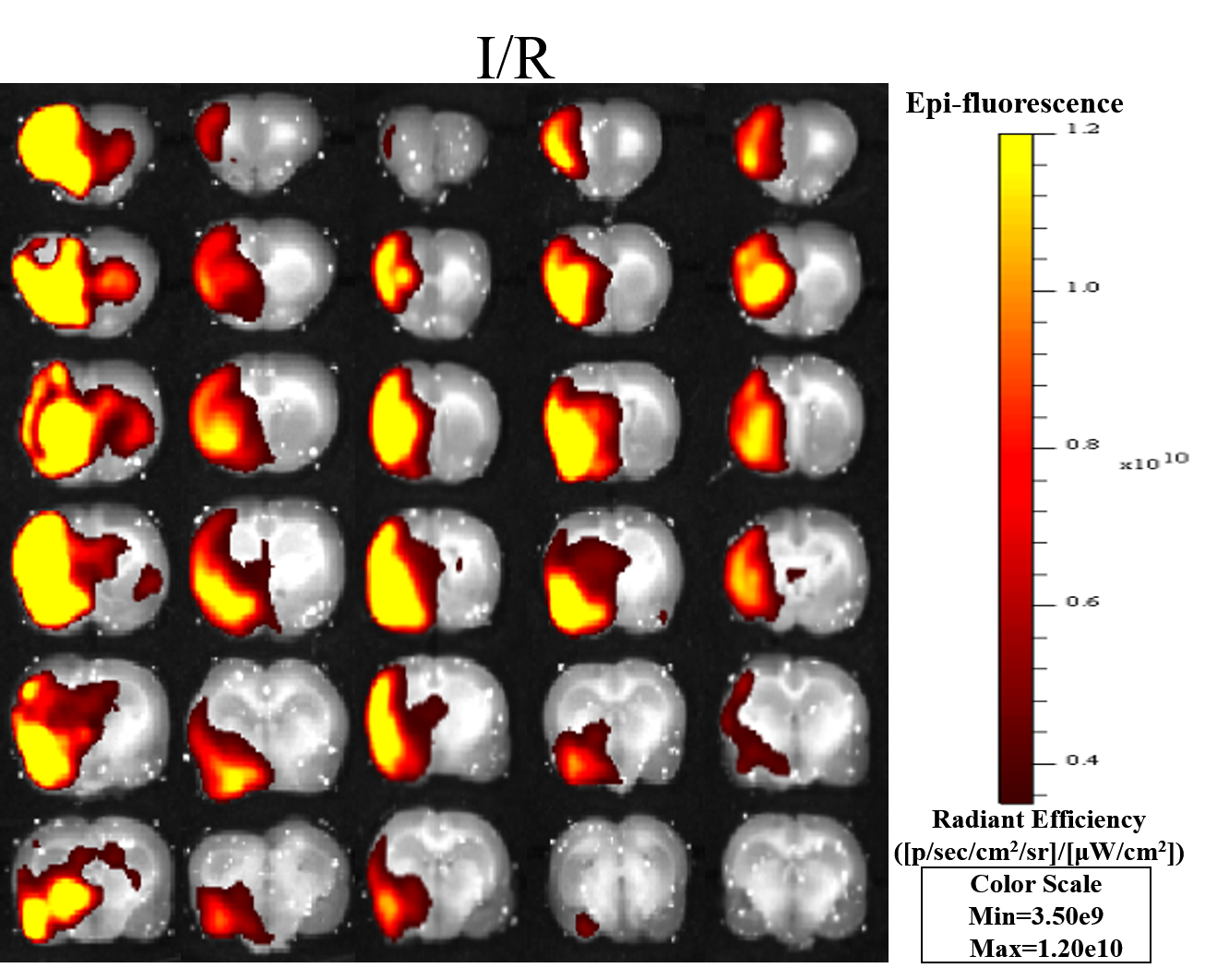

Supplement: Supplementary file 2 [file datasheet2.zip › IVIS-Model.tif]

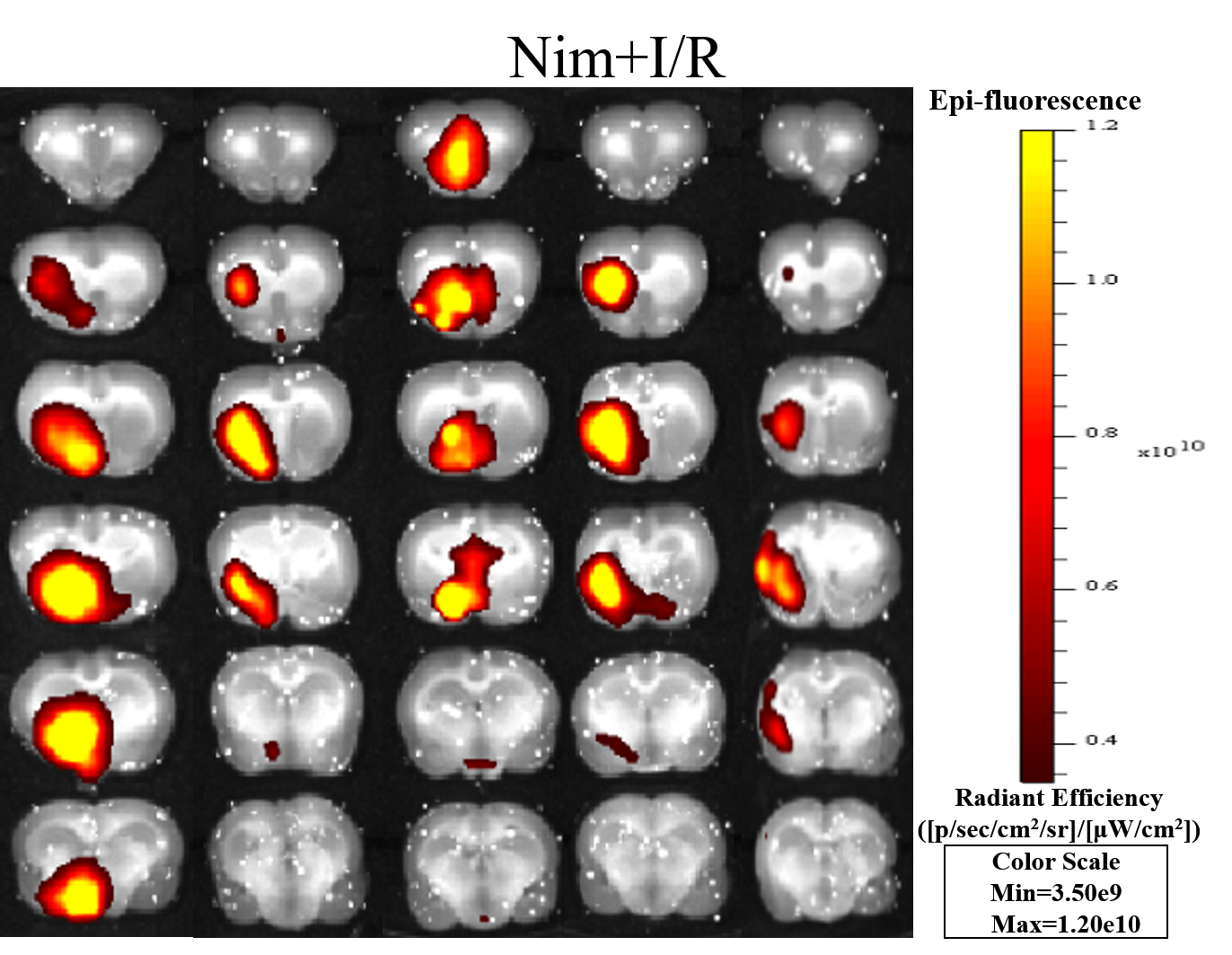

Supplement: Supplementary file 2 [file datasheet2.zip › IVIS-Nim.tif]

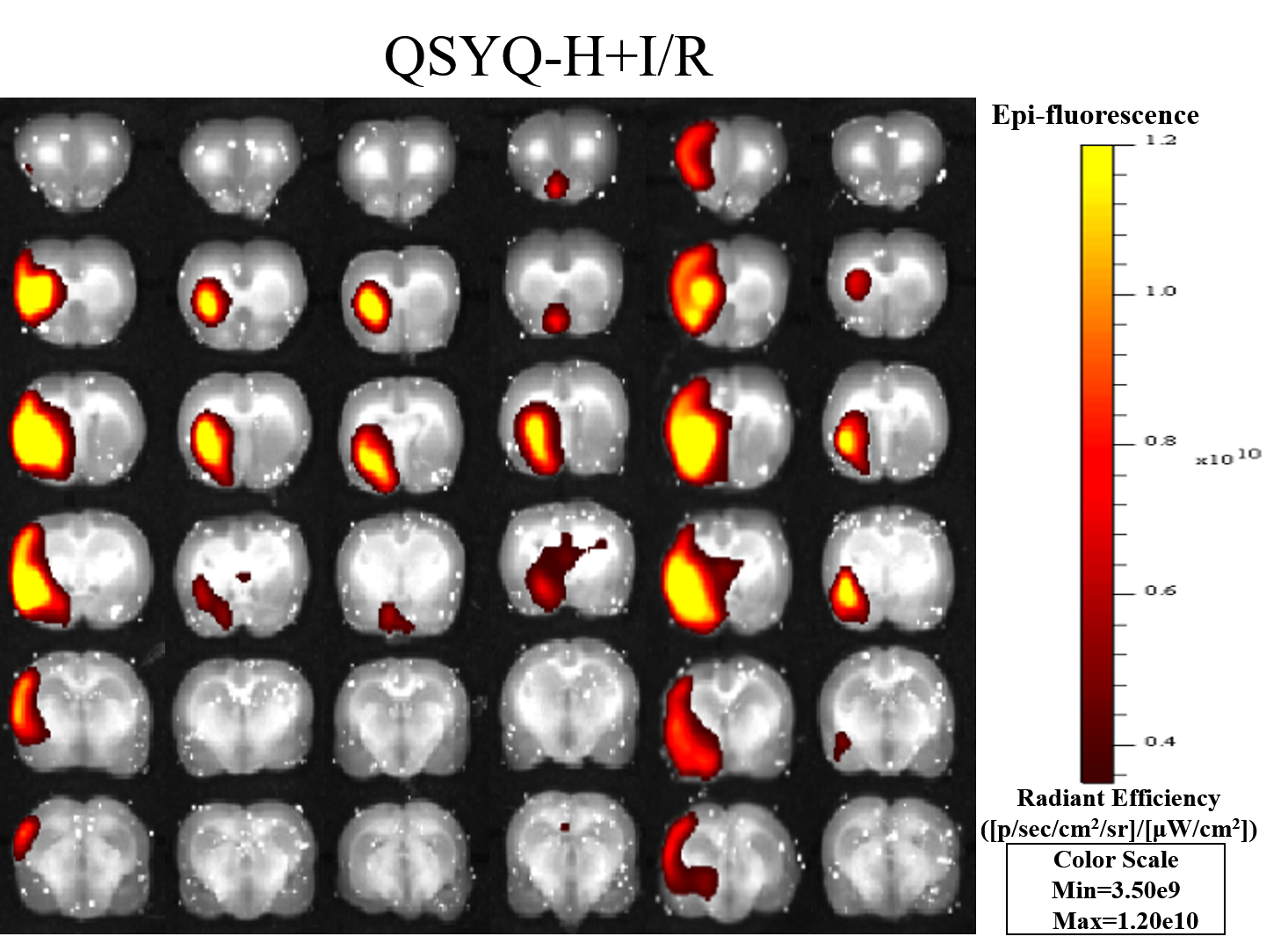

Supplement: Supplementary file 2 [file datasheet2.zip › IVIS-QSYQ-H.tif]

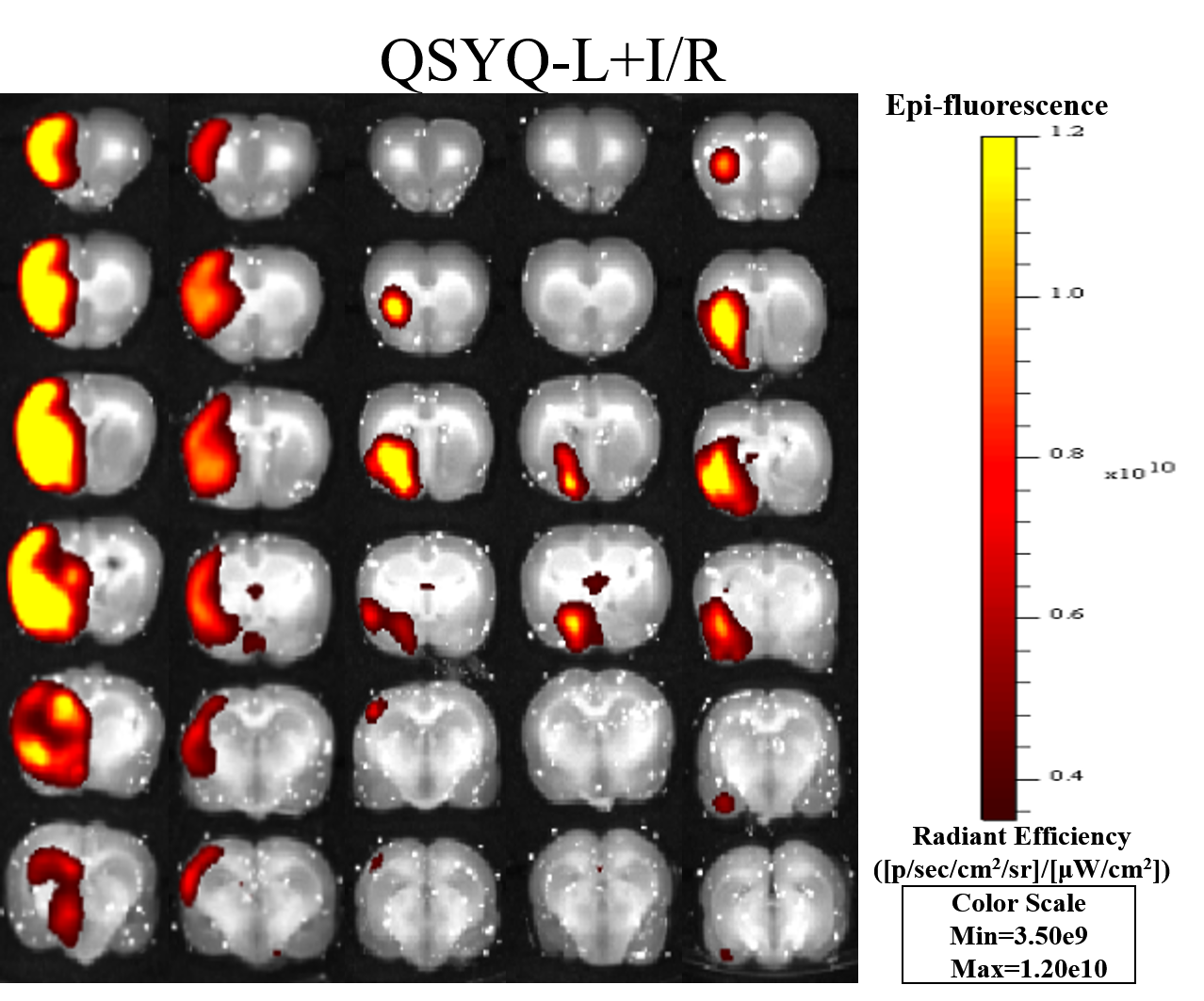

Supplement: Supplementary file 2 [file datasheet2.zip › IVIS-QSYQ-L.tif]

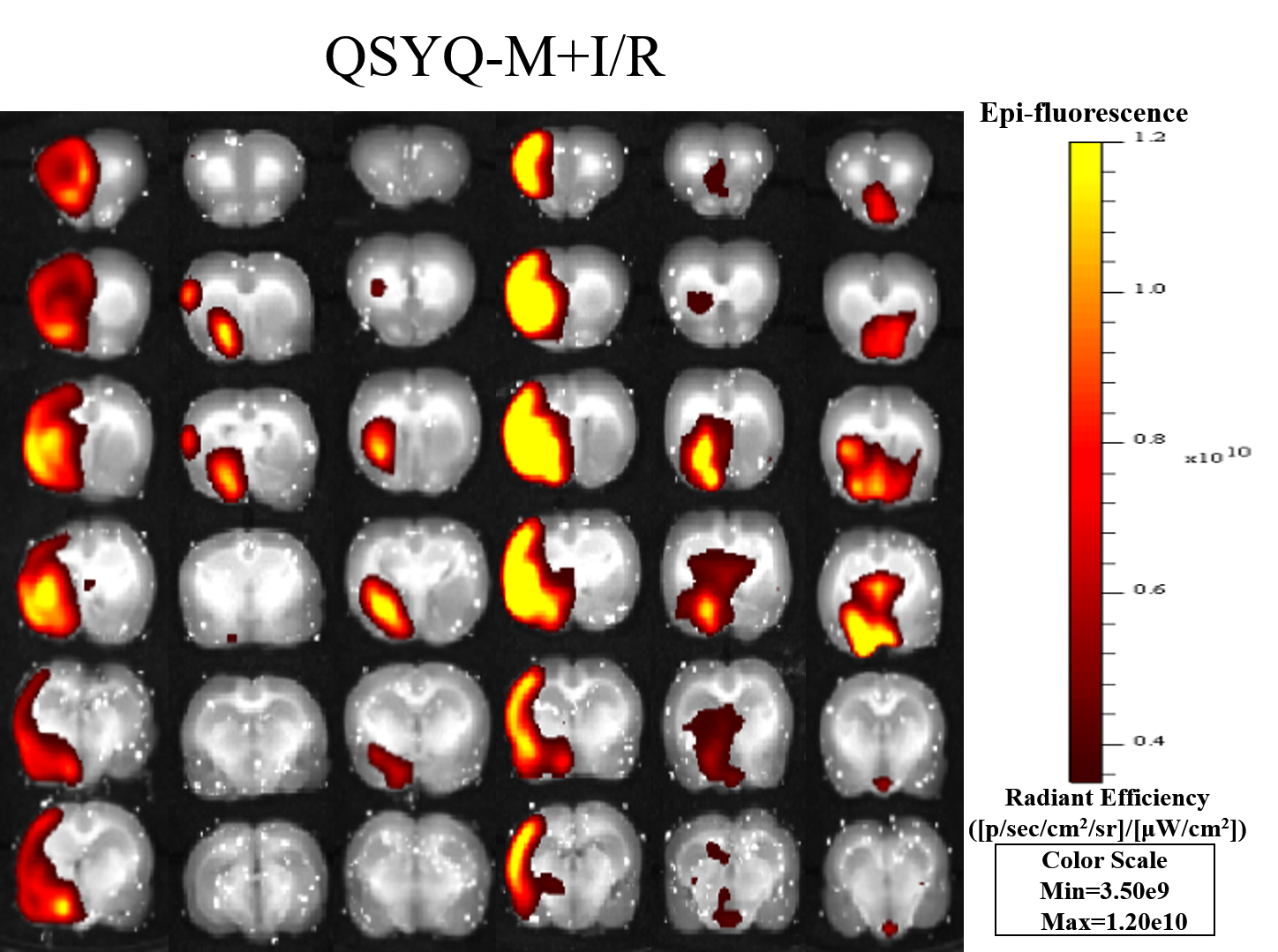

Supplement: Supplementary file 2 [file datasheet2.zip › IVIS-QSYQ-M.tif]

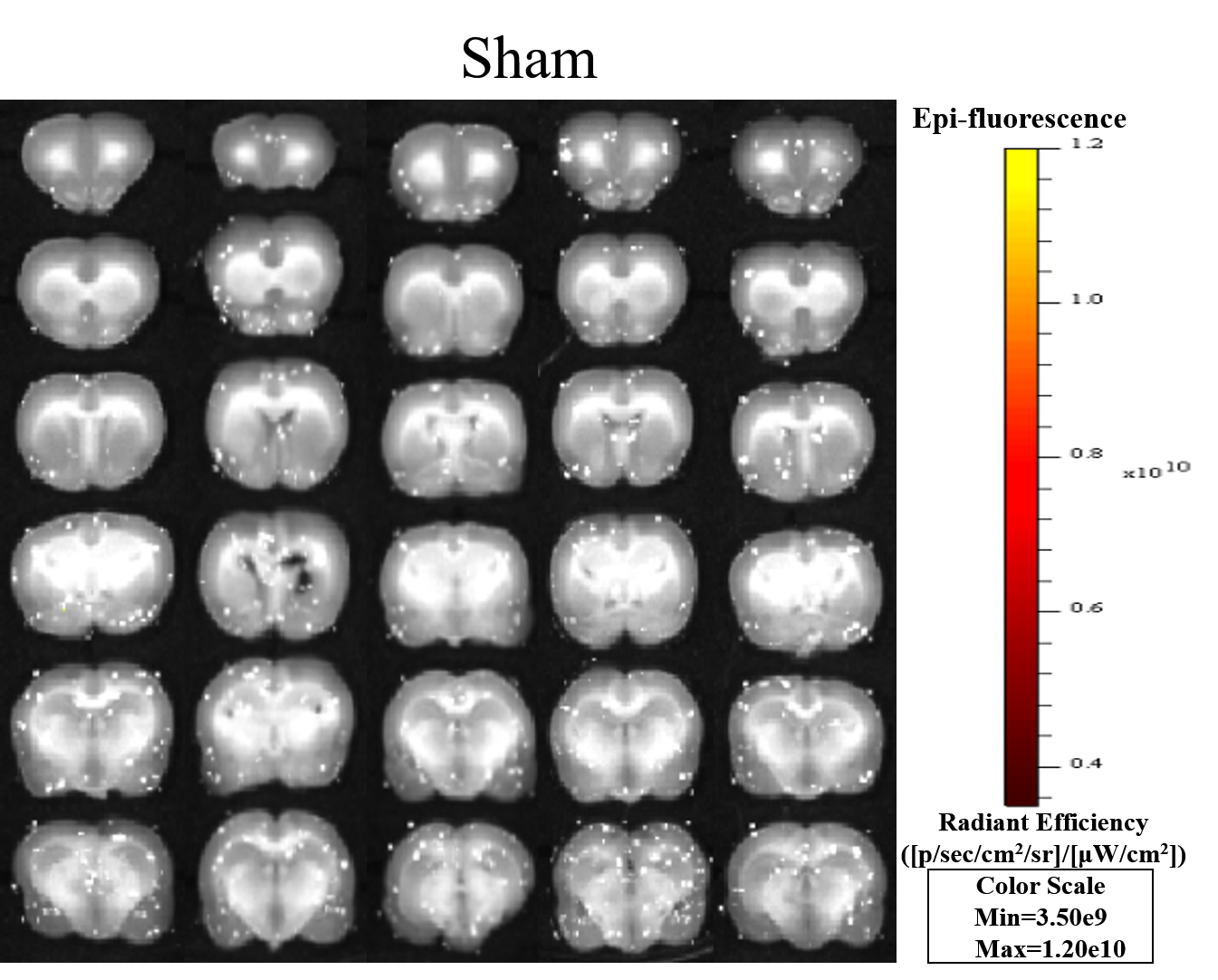

Supplement: Supplementary file 2 [file datasheet2.zip › IVIS-Sham.tif]

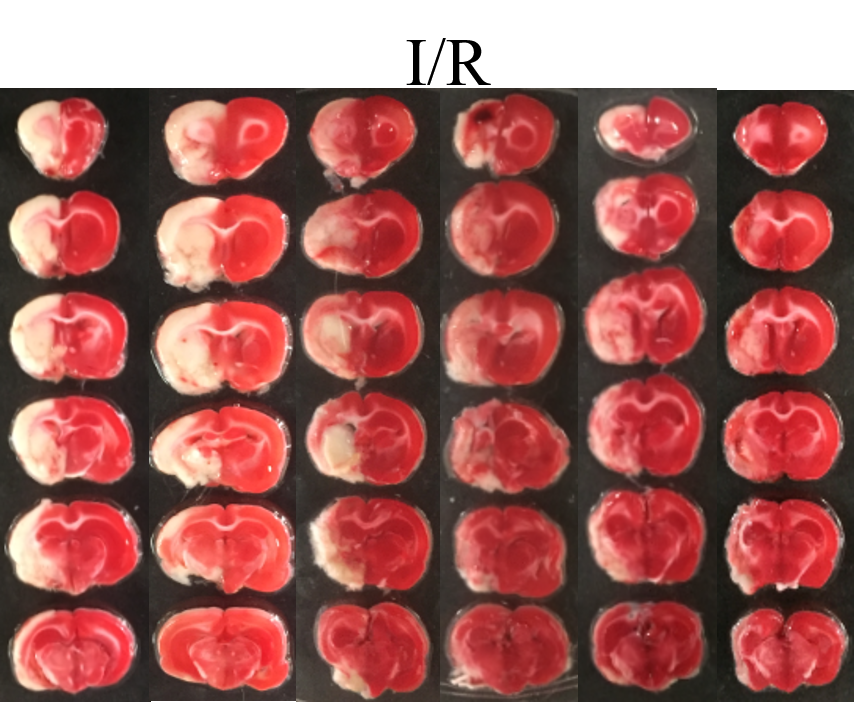

Supplement: Supplementary file 2 [file datasheet2.zip › TTC-Model.tif]

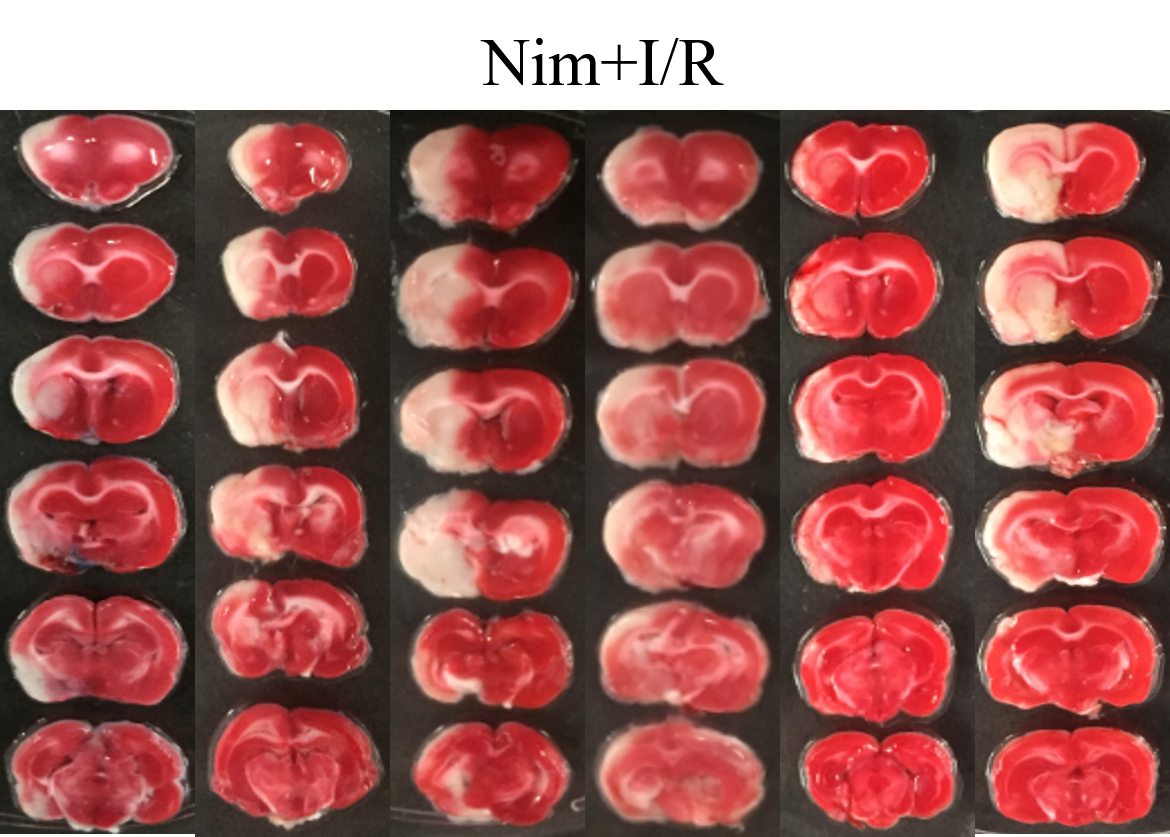

Supplement: Supplementary file 2 [file datasheet2.zip › TTC-Nim.tif]

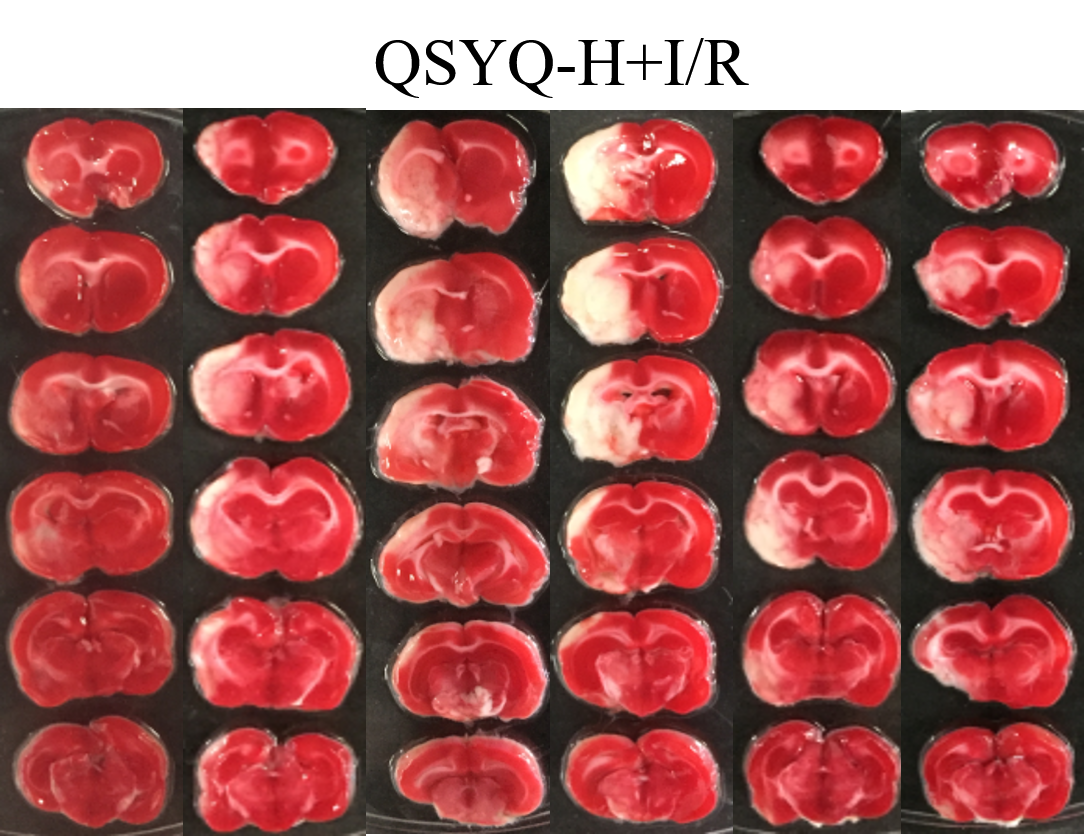

Supplement: Supplementary file 2 [file datasheet2.zip › TTC-QSYQ-H.tif]

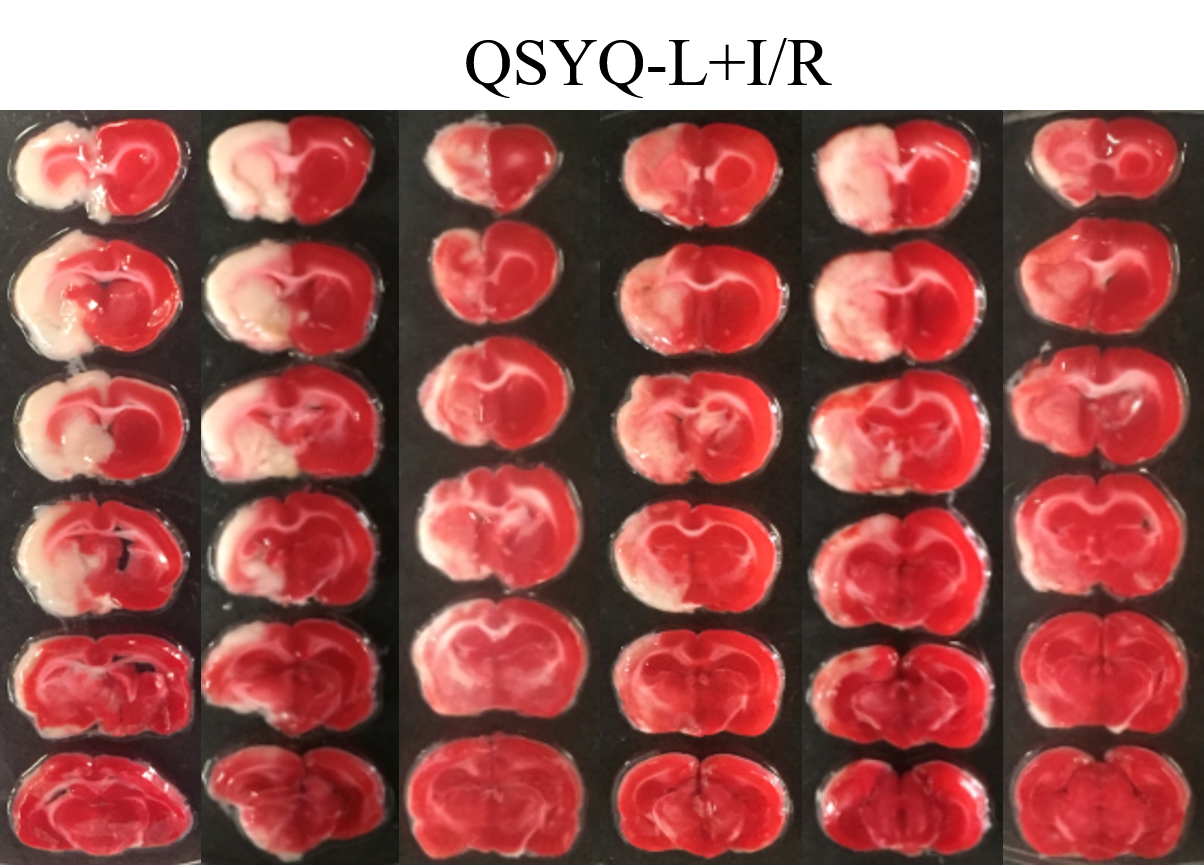

Supplement: Supplementary file 2 [file datasheet2.zip › TTC-QSYQ-L.tif]

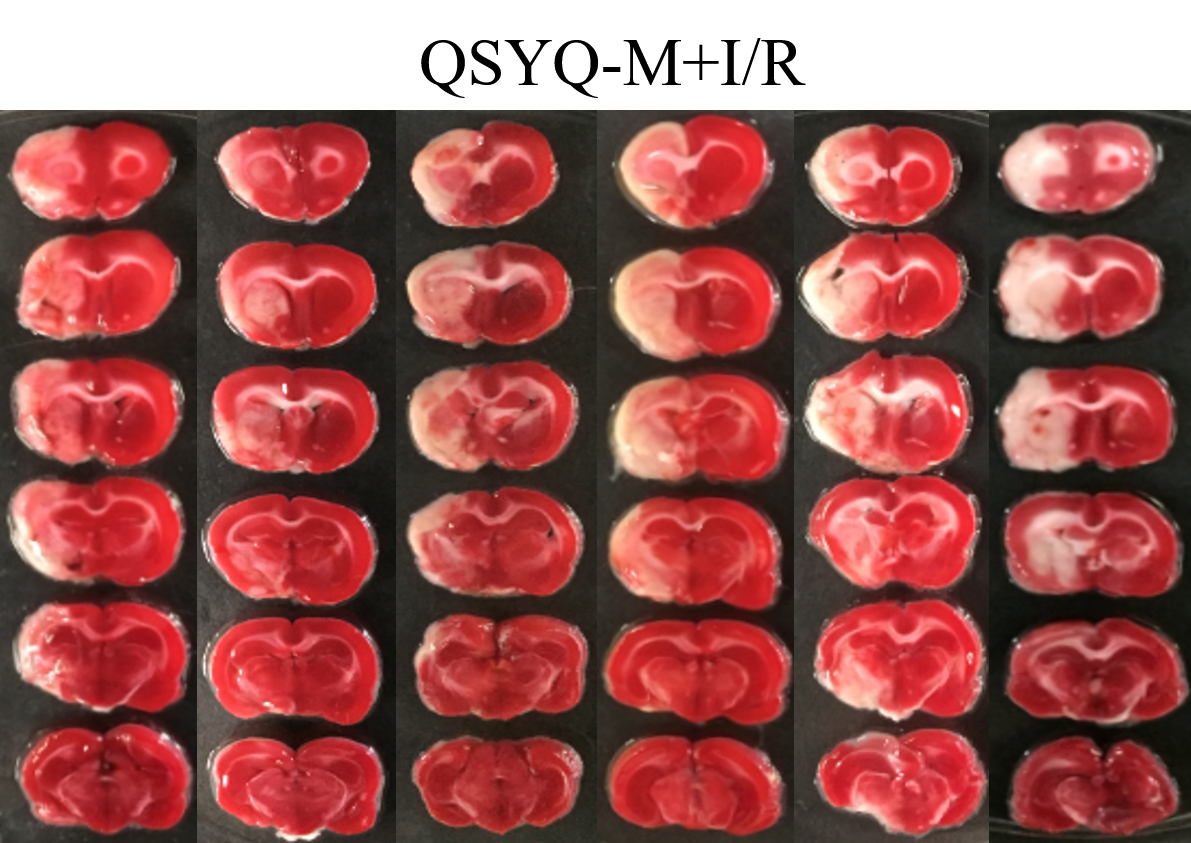

Supplement: Supplementary file 2 [file datasheet2.zip › TTC-QSYQ-M.tif]

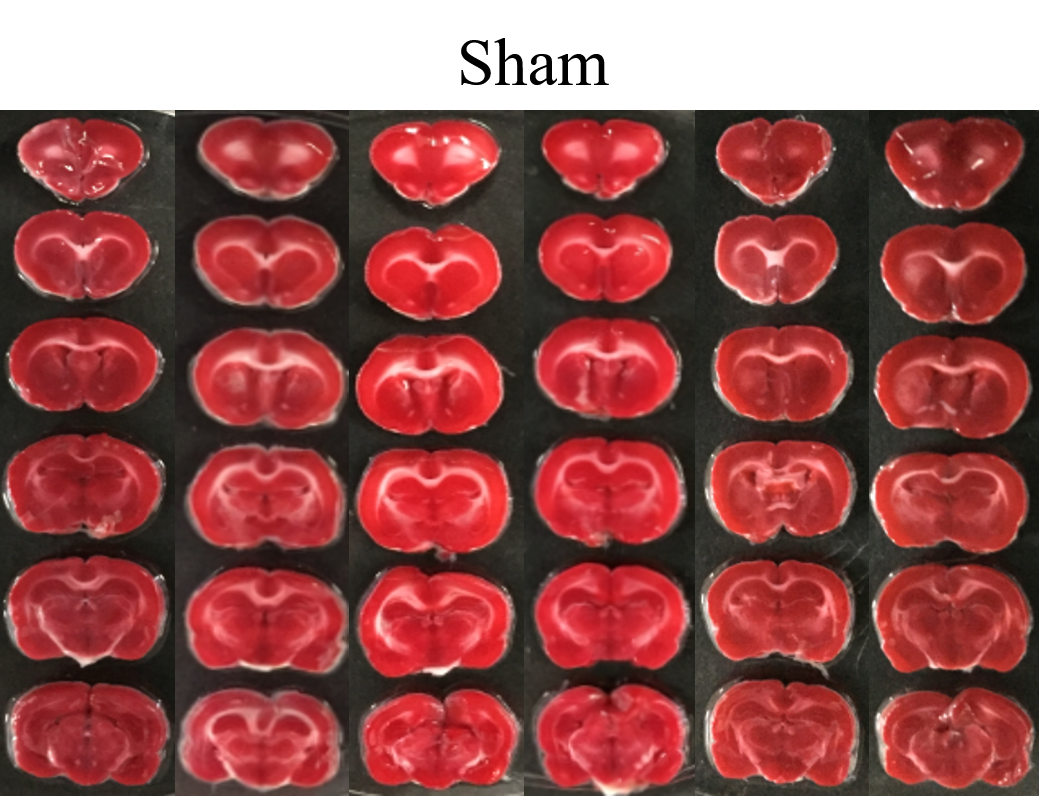

Supplement: Supplementary file 2 [file datasheet2.zip › TTC-Sham.tif]

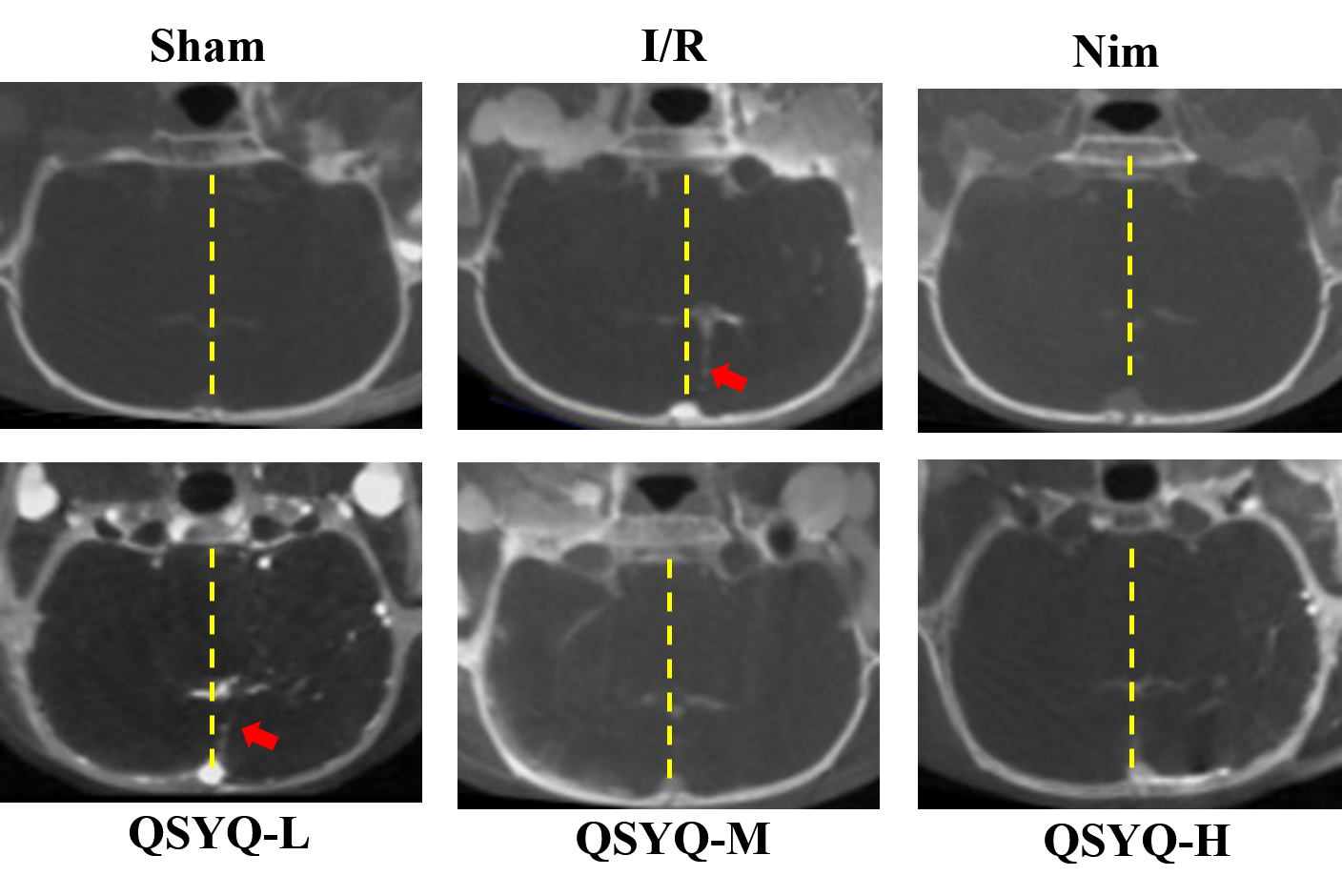

Supplement: Supplementary file 2 [file datasheet2.zip › cerebral edema.tif]
