# Supplementary material for: Galectin-3 Mediated Inflammatory Response Contributes to Neurological Recovery by QiShenYiQi in Subacute Stroke Model
Source: Front Pharmacol. 2021 Apr 19;12:588587. doi: 10.3389/fphar.2021.588587 (PMC8089377; doi:10.3389/fphar.2021.588587)
Supplement: Supplementary file 2 [file datasheet2.zip › GO analysis.docx]

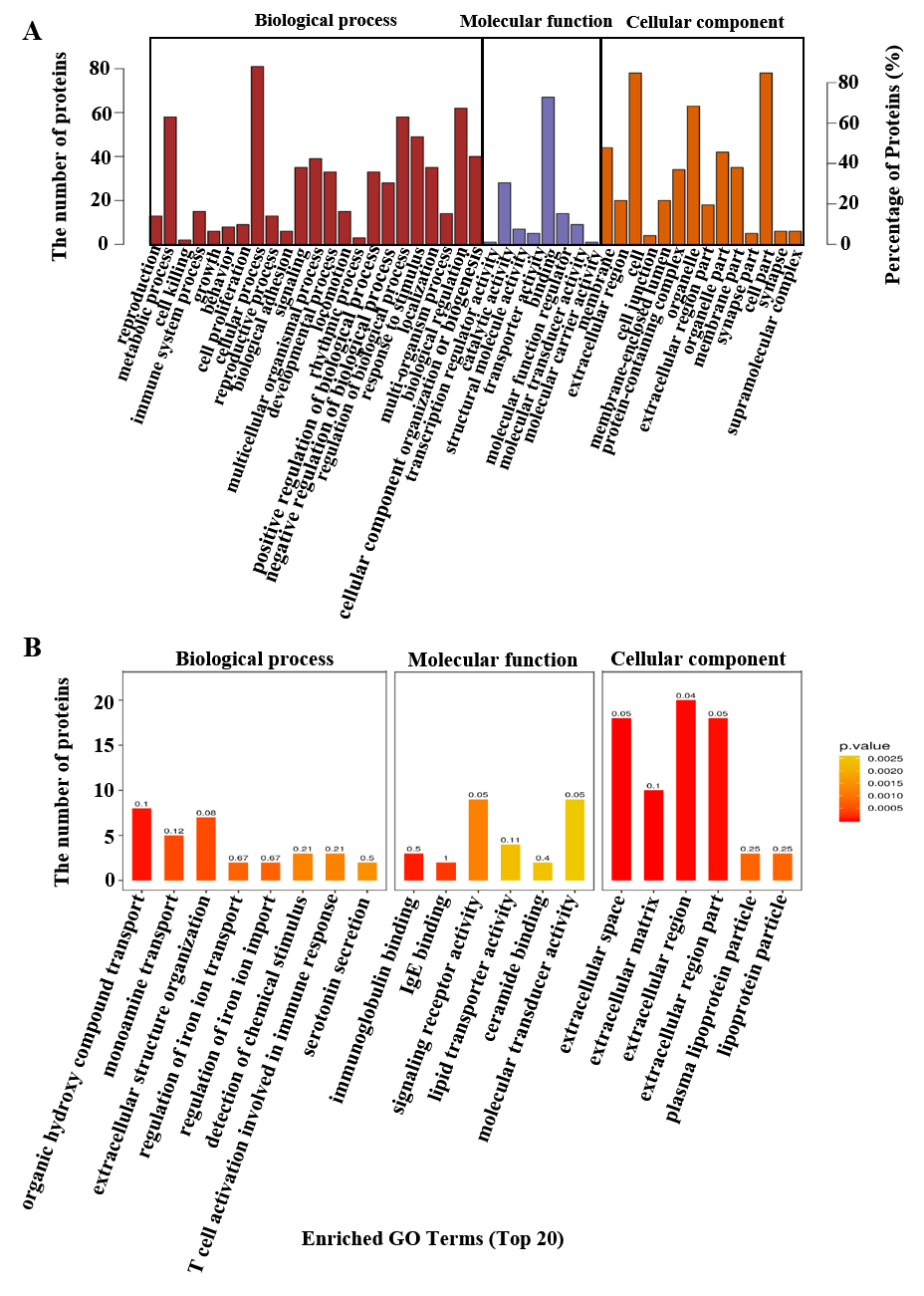


**Figure . GO analysis and functional enrichment analysis of differentially expressed proteins.** (A) GO functional annotation analysis and classification. The 92 differentially expressed proteins were categorized into biological process, molecular function, and cellular component in accordance with GO annotation. (B) GO functional enrichment analysis. The enrichment analysis for GO on three ontologies (biological process, molecular function, and cellular component) was performed based on the Fisher’s exact test with the enrichment score of *p*-value (with a decreasing scale colored from yellow to red).
