# Supplementary material for: Galectin-3 Mediated Inflammatory Response Contributes to Neurological Recovery by QiShenYiQi in Subacute Stroke Model
Source: Front Pharmacol. 2021 Apr 19;12:588587. doi: 10.3389/fphar.2021.588587 (PMC8089377; doi:10.3389/fphar.2021.588587)
Supplement: Supplementary file 2 [file datasheet2.zip › WB data.docx]

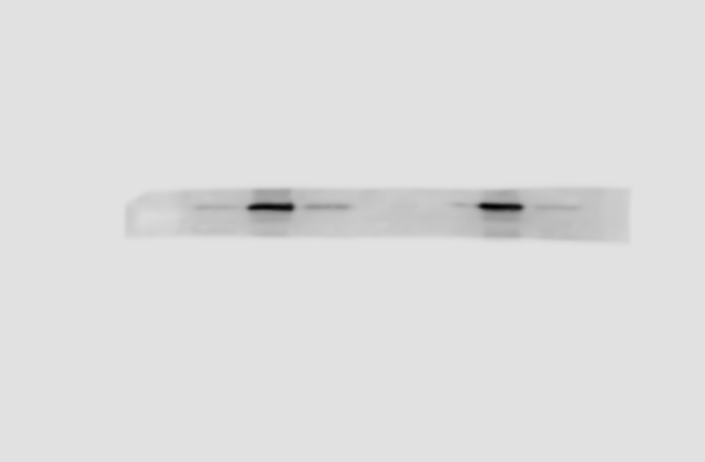


Galectin-3

Sham Model QSYQ


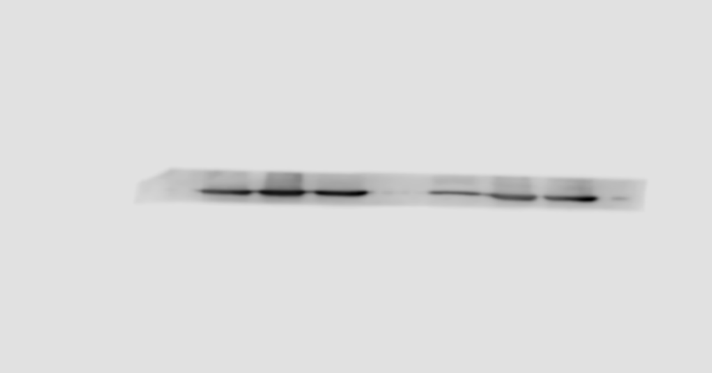


β-actin

Sham Model QSYQ


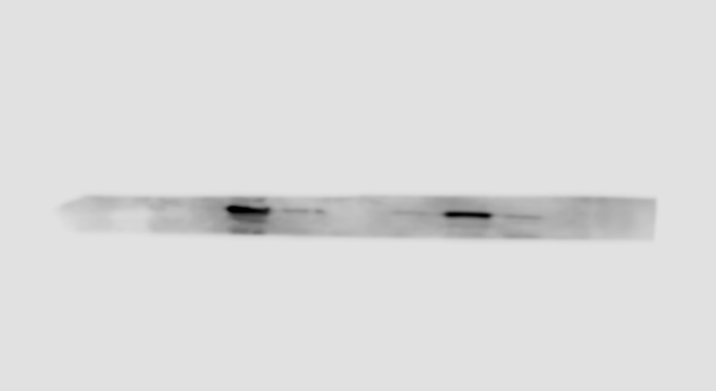


Sham Model QSYQ

Sham Model QSYQ

Galectin-3


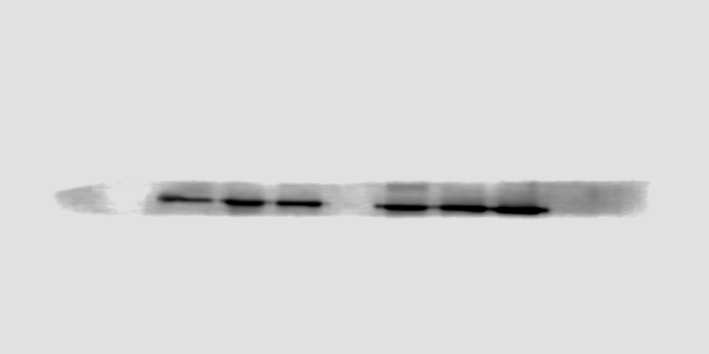


β-actin

Sham Model QSYQ

Sham Model QSYQ
